# To learn or not to learn? Model-based estimation of motor learning in a large, “in the wild”, home rehabilitation database

**DOI:** 10.1371/journal.pdig.0001598

**Published:** 2026-08-27

**Authors:** Yan Wen, Dongze Ye, Rukshana Poudel, Dan Zondervan, David Reinkensmeyer, Nicolas Schweighofer

**Affiliations:** 1 Department of Computer Science, University of Southern California, Los Angeles, California, United States of America; 2 Department of Biokinesiology and Physical Therapy, University of Southern California, Los Angeles, California, United States of America; 3 Flint Rehab, Irvine, California, United States of America; 4 Department of Mechanical and Aerospace Engineering, University of California at Irvine, Irvine, California, United States of America; Maastricht University Cardiovascular Research Institute Maastricht: Universiteit Maastricht Cardiovascular Research Institute Maastricht, NETHERLANDS, KINGDOM OF THE

## Abstract

Motor learning, defined as practice-related processes leading to relatively permanent changes in response capability, is critical for regaining motor function following brain injury. However, quantifying learning in real-world, unsupervised settings remains challenging. Here, we present a model-based framework for estimating the presence and extent of motor learning across multiple tasks and users from a large “in-the-wild” rehabilitation dataset collected via the FitMi sensor system. We analyzed 4,661 episodes from 398 users practicing 20 upper-limb tasks for at least 50 sessions. For each episode, we modeled the effect of daily dose (repetitions) on performance (repetitions per second) using a discrete-time first-order state-space model, in which a latent motor memory evolves through cumulative practice. The model employed a learning rate for memory updates and a forgetting time constant for decay. Simulation-based recovery confirmed the robustness of this procedure despite realistic noise and irregular practice schedules. We utilized a likelihood ratio test (LRT) to compare this learning model against a non-learning null model. Overall, 38.3% of episodes were classified as learning. Among these, 44.6%, which correspond to 17.1% of all episodes, exhibited a time constant exceeding 30 days, consistent with durable motor memory. In contrast, non-learning episodes exhibited short time constants (under 2 days), reflecting transient fluctuations. Task-level analysis revealed no clear dichotomy between learnable and non-learnable tasks. Among users practicing at least five tasks, 18% were non-learners and only 5% exhibited learning on all tasks, suggesting that generalizable improvements were rare. Our findings demonstrate that real-world rehabilitation induces detectable motor learning in specific tasks and users. Future research will incorporate user-level covariates, characterize higher-order learning dynamics, and validate findings against clinical outcomes to establish a link with functional recovery. These results offer a path toward optimizing post-stroke recovery through individualized task selection.

## Author summary

Stroke survivors often require long periods of arm and hand therapy to regain movement. Home rehabilitation with devices like the FitMi enables high doses of practice at a reduced cost. However, it has remained unclear whether such unsupervised practice leads to long-lasting motor skill learning or simply transient performance changes. We analyzed thousands of long-term home-exercise records from nearly 400 FitMi users. By applying a computational model that separates lasting motor memory from temporary improvements, we found that only about one-third of user-task episodes showed clear evidence of motor learning. Furthermore, fewer than one in five users exhibited learning that persisted for more than a month. These findings suggest that sustained motor memory formation during home-based rehabilitation may be less common than previously assumed. Our approach provides a framework for therapists to design personalized motor therapy programs and can guide future adaptive digital health tools that tailor exercises to each individual’s unique learning potential.

## Introduction

Stroke survivors with persistent upper extremity (UE) impairment often experience reductions in independence in daily activities, quality of life, and ability to return to productive engagement [1,2]. Intensive UE rehabilitation can reduce long-term impairment after stroke. Although the exact dose-response relationship is unknown, recovery appears to depend on the overall dose of movement practice [3–6]. The prevailing rehabilitation paradigm is based on intensive in-clinic therapy [7], which can be costly, time-intensive, and geographically inaccessible for numerous patients. In addition, the actual dose of therapy delivered in clinical settings is frequently low [8,9], which can lead to suboptimal recovery outcomes. Consequently, there is a crucial need for effective, personalized home-based rehabilitation solutions that support long-term recovery.

Home therapy devices such as the FitMi [10] provide interactive, game-based exercise for both upper and lower extremities. The devices can provide a high dose of task-oriented training at a relatively low cost and can be effective in reducing UE impairment [11].

Improvements resulting from task practice, such as during FitMi training, are at least partly due to motor learning. Indeed, motor learning, a set of internal processes associated with practice that leads to relatively permanent changes in skilled behavior, is considered to be critical for regaining motor function following brain injury [12]. As individuals, whether they have a neurological injury or not, engage in practice, their performance improves through skill acquisition [13]. This learning is dependent on neural plasticity in both the healthy and lesioned brain. While improvements during the chronic phase are often attributed to the development of compensatory techniques, progress during the earlier post-stroke phase appears more rapid and may generalize through a genuine reduction in impairment [14–17].

Despite this theoretical potential for recovery through motor learning, quantifying motor learning in real-world, unsupervised rehabilitation settings remains challenging. For instance, during FitMi practice, it is unclear whether motor performance improves over the long term, for which users, and for which tasks.

Here, we present a model-based framework to estimate the presence and, if applicable, the extent of motor learning across multiple tasks and users from a large “in-the-wild” rehabilitation dataset collected via FitMi, a home rehabilitation device.

Because recovery of function post-stroke and motor learning involve changes that are initially fast and then gradual, and exhibit both improvements and decrements in performance levels, a dynamical, state-space systems approach provides a useful modeling framework. For instance, state-space models have been widely used to model motor adaptation [18,19], including post-stroke [20,21], with models that include one or two internal states. Although there is less work on modeling motor skill learning, especially in post-stroke populations, we have previously modeled the overall effect of task practice on recovery using a state-space model approach [22]. This prior body of work has focused on laboratory-based learning tasks with controlled practice schedules. The originality of the present study lies in applying a latent state-space model to quantify motor learning across thousands of real-world episodes from hundreds of users with heterogeneous, self-directed practice patterns in an unsupervised “in the wild” home setting—a scale and context that has not been systematically studied with computational modeling approaches. For this, we assumed a simple one-dimensional dynamical model with both a nonlinear learning component, driven by the dose of practice, and a forgetting component that allows for the decay of motor function following practice.

Specifically, we expand the application of state-space modeling across a wide range of FitMi exercises, guided by the following observations in motor skill learning [13]: (1) increasing the amount of practice increases efficacy (“practice makes perfect”), (2) increasing the amount of practice decreases efficiency (“the diminishing returns of practice”), and (3) performance decays following practice. We thus model motor memory as a function of a sublinear effect of dose in the current time step and a forgetting term that depends on the memory at the previous time step and leads to decay. In line with the state-space approach, current performance is the weighted sum of this memory and a “memoryless” term representing the immediate effect of practice. Using this model, we estimate motor learning independently for each user and each FitMi task that the user practiced for at least 50 days (which we define as an episode). We then compare the model fit to that of a model without the memory term. This allows us to classify each episode as learning versus non-learning. Finally, we characterize learning episodes via their estimated parameters (e.g., forgetting time constant τ, learning rate, immediate-effect magnitude) and systematically contrast their training schedules—dose, duration, and variability—with those of non-learning episodes. In sum, this study extends prior state-space paradigms by adapting the framework specifically for skill learning, handling irregular real-world schedules, and enabling large-scale, individualized parameter estimation across heterogeneous users and tasks.

## Methods

### FitMi overview

**Fig 1:**
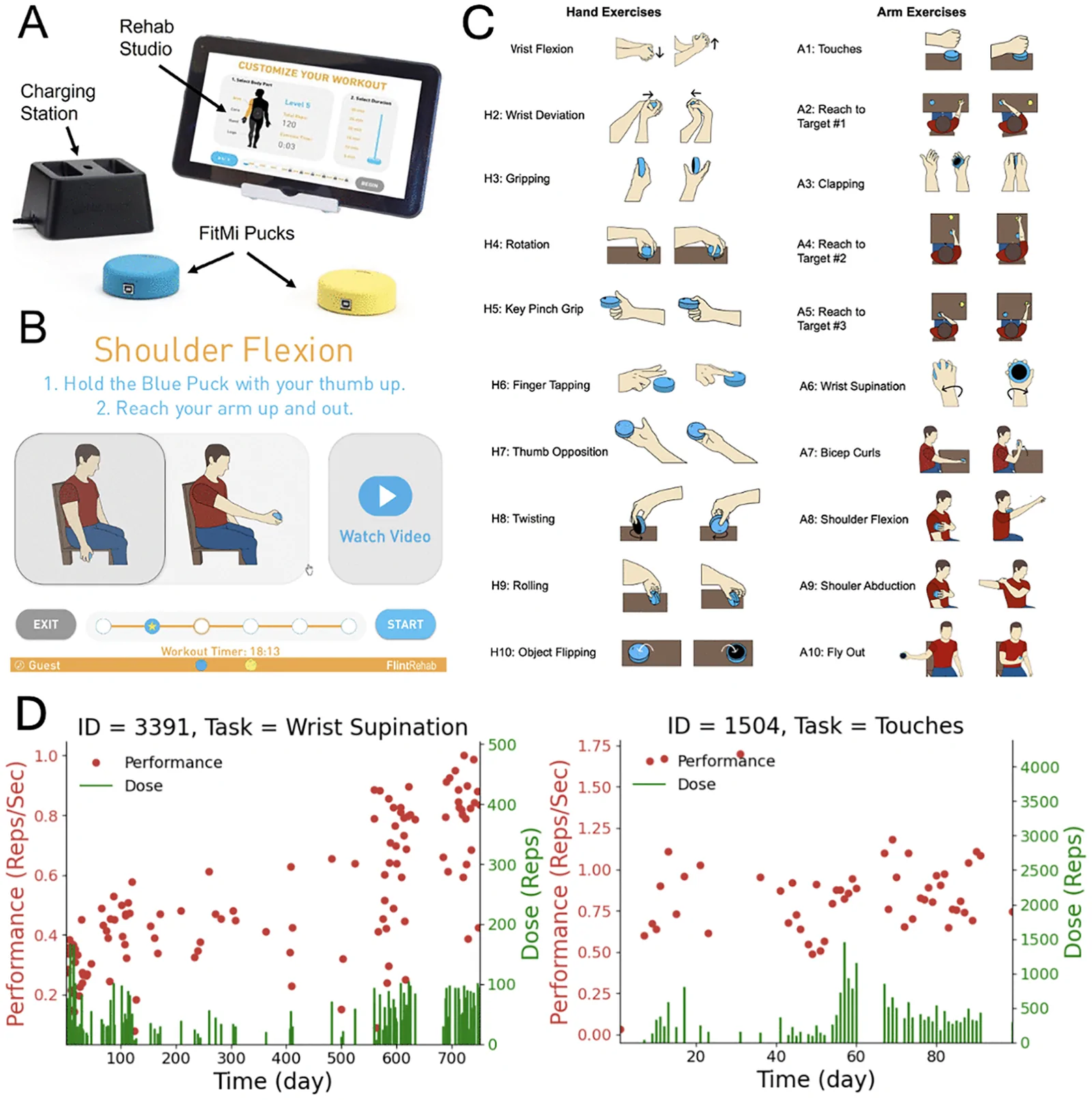
FitMi system and examples of dose and performance data extracted from the database. A) The FitMi system includes two force- and motion-sensing pucks, a charging station, and the RehabStudio software application for gamified exercise. B) Example of an arm exercise (shoulder flexion) as presented in RehabStudio. C) The 20 exercises supported by FitMi, with 10 targeting the hand and 10 targeting the arm. At each session, users freely select tasks to practice. Thus, due to the unsupervised nature of home use, task schedules (timing, frequency, and dose) vary widely across users. D) Examples of time-series data from two representative episodes for two users. In both panels, the red points indicate observed performance (repetitions per second; left axis), and the green vertical lines represent input dose (number of repetitions per day; right axis). The left panel illustrates an episode with longer duration and more training sessions; the right panel shows an episode with shorter duration and fewer training sessions. Note that performance is only observed on days with non-zero dose input, illustrating the sparse and irregular nature of real-world home rehabilitation.

The FitMi [23] is a commercially available, FDA-listed medical device designed to support self-directed movement rehabilitation for individuals with neurological impairments, such as stroke survivors. The system enables high-dose, gamified home exercise without direct therapist supervision and has been shown to improve motor function in clinical studies (Fig 1A, B). The FitMi system includes two wireless pucks with embedded sensors that detect both force and motion, and an optional silicone strap to assist users with impaired hand function. The accompanying software application delivers interactive, therapist-designed exercises. For this study, we focused exclusively on the 20 upper-limb exercises—10 for the hands and 10 for the arms—illustrated in Fig 1C.

Each exercise is a timed, repetition-based task. Users were free to choose which tasks to practice. For each task, users are instructed to perform a series of repetitions between defined start and end positions, aiming to achieve a target number of repetitions within a specified time frame. A leveling system progressively increases challenge by raising repetition targets or reducing time limits following successful completion of an exercise, while also unlocking new exercises as progress continues. This gamified structure encourages engagement and progressive training. The system also provides real-time visual feedback and tracks performance history across sessions. After each session, anonymous sensor data and user program interactions are automatically uploaded to a secure cloud database managed by Flint Rehabilitation Devices.

### Data preprocessing

This study used data obtained from a commercial product and is based on the secondary use of anonymized data. When individuals purchase the device, they sign an agreement allowing their data to be collected to help improve the system. The data are recorded in an anonymized format. The research team does not have access to any information linking the FitMi user to the data (this would require the users to send a code located on their computer). This study is therefore exempt from ongoing Institutional Review Board (IRB) oversight; see, for instance, question 14 on research involving human subjects on the following NSF website [24].

We used the FitMi dataset available on 06/16/2023, which contains usage logs from 7,883 users. This “in-the-wild” dataset includes fine-grained behavioral records for each training session. Each row in the raw dataset corresponds to a single user-task training session. To prepare the data for analysis and modeling, we focused on five key fields: *uid*: anonymized user identifier; *task*: name of the performed exercise; *t_created*: timestamp marking the session creation; *reps*: total number of repetitions completed during the session; and *time_spent*: total time (in seconds) spent in the session.

These features provide a granular view of unsupervised home practice, capturing input dose as the number of repetitions and output performance as the number of repetitions per second. We used these detailed behavioral data to enable model-based characterization of motor learning versus performance for each user-task episode.

*Data pre-processing and discretization.* All data were discretized by calendar day to establish a fixed temporal structure with a time step manageable for modeling. Thus, for each (user, task) episode, all repetitions performed on the same day were aggregated into a single time point (day), with the total number of repetitions (*sum_reps*) and the total time spent in seconds (*sum_time_spent*). This yielded a daily time series starting from day 1 for each episode. Day 1 is typically a different calendar day for each task, including for the same user practicing multiple tasks.

*Episode Segmentation and Filtering.* The dataset was segmented into episodes, where each episode corresponds to one user repeatedly practicing one specific task. Episodes were retained only if they contained at least 50 unique daily sessions, ensuring sufficient temporal resolution for learning analysis.

*Assessment of Motor Performance*. On each day of a (user, task) episode, the output performance was computed as the ratio of aggregated repetitions to the aggregated time spent (i.e., *sum_reps/sum_time_spent*), yielding task performance in units of repetitions per second. This metric captures the efficiency with which users complete task repetitions and serves as a practical proxy for task-level motor performance during training.

*Normalization of Input Dose.* Because the number of target repetitions varies across tasks of different complexity and challenge levels, we normalized the daily input dose within each task by dividing it by the 95th percentile of that task’s daily dose distribution across all users. This approach preserved relative variability within each task while reducing scale differences across tasks, enabling meaningful comparisons of learning rates across tasks in modeling analysis (see below).

Fig 1D illustrates time-series data from two representative (user, task) episodes. Daily input doses are visualized as green vertical lines, and observed performance is represented by red points. The two examples in Fig 1D show episodes with high versus low engagement: the left example spans a longer duration but involves less frequent training: 751 days with 120 training sessions, averaging one session every 6.3 days. In contrast, the right example is much shorter but reflects denser practice: 99 days with 54 training sessions, averaging one session every 1.8 days. We note that because output performance was recorded only on days when repetitions were performed, the time series are often temporally sparse.

### Modeling motor learning with state-space models

#### The learning model.

To quantify motor learning over time, we modeled the input–output behavior of each rehabilitation episode using a first-order, discrete-time, stochastic state-space model. In keeping with the state-space framework [25], the model outputs reflect both long-term learning effects, represented by an internal memory state that evolves with practice, and short-term performance fluctuations, driven by immediate input (i.e., same-day practice).

Specifically, for each user *i* and task *k*, we define a latent motor memory state $X_{ik}$ that accumulates with practice and decays over time. The model consists of the following state transition and observation equations:


$$\begin{array}{c} \begin{array}{c} X_{ik}(t+\text{1})=A_{ik}*X_{ik}(t)+B_{ik}*{\left(U_{ik}(t)\right)}^{p_{\text{1}}}+{pn}_{ik}(t) \end{array} \end{array}$$ (1)

$$\begin{array}{c} \begin{array}{c} Y_{ik}(t)=C_{ik}*X_{ik}(t)+D_{ik}*{\left(U_{ik}(t)\right)}^{p_{\text{2}}}+{mn}_{ik}(t) \end{array} \end{array}$$ (2)

where:

- $X_{ik}(t)\geq \text{0}$: latent motor memory at day *t*,
- $U_{ik}(t)\geq \text{0}$: normalized input dose (repetitions) on day *t*,
- $Y_{ik}(t)\geq \text{0}$: observed performance (repetitions per second) on day *t*,
- $A_{ik}=e^{-\frac{\text{1}}{\tau_{ik}}}$: memory retention factor, derived from the forgetting time constant $\tau_{ik}>\text{0}$.
- $B_{ik}\geq \text{0}$: learning rate, governing how input modifies memory,
- $C_{ik}\geq \text{0}$: output gain, translating memory into performance,
- $D_{ik}\geq \text{0}$: feedthrough gain, modeling immediate input effects,
- $p_{\text{1}},\;p_{\text{2}}\in (\text{0},\;\text{1})$: fixed sublinear exponents shared across all episodes,
- ${pn}_{ik}(t)\;\sim N\left(\text{0},\;Q_{ik}\right)$: process noise on day *t*,
- ${mn}_{ik}(t)\;\sim N\left(\text{0},\;R_{ik}\right)$: measurement noise on day *t*.
- The initial memory state is unknown and modeled as a Gaussian prior:$\;X_{ik}(\text{1})\;\sim \;N\left(X_{\text{1},\;ik},\;V_{\text{1},\;ik}\right)$.

Note that this full model enables the separation of learning (through memory: $X_{ik}(t)$) from immediate performance fluctuations (through the input feedthrough term $D_{ik}*{\left(U_{ik}(t)\right)}^{p_{\text{2}}}$ in Eq. 2).

The two main parameters of interest were the learning rate $B_{ik}$, which updates the motor memory, and the forgetting time constant $\tau_{ik}>\text{0}$, which directly relates to the retention factor $A_{ik}$, and controls how quickly the internal memory decays in the absence of input. Rather than estimating $A_{ik}$ directly, we reparameterized the model using the time constant $\tau_{ik}$, measured in days: $\tau_{ik}=-\frac{\text{1}}{\text{ln}A_{ik}}$. This formulation is consistent with prior literature on motor memory retention [26] and allows for a direct interpretation of retention as the characteristic time scale of forgetting; it also avoids poor estimation when the retention factor is close to 1. By definition, τ represents the time required for the internal memory to decay to about 37% of its initial value (i.e., a 63% loss of retention), a convention used in motor-memory studies [26]. Higher values of $\tau_{ik}$ indicate slower decay and better long-term retention.

In motor rehabilitation, learning exhibits diminishing returns: larger doses of practice lead to lower efficiency (i.e., gains per unit dose) of practice [27], resulting in a sublinear dose-response relationship. Here, we assumed (and confirmed in our results) that this nonlinearity also occurs within a single day of practice and applied fixed sublinear exponents $p_{\text{1}},\;p_{\text{2}}\in (\text{0},\;\text{1})$ to the input dose term $U_{ik}(t).$ This transformation yields a sublinear, concave relationship between dose and response, capturing diminishing efficiency. The exponents $p_{\text{1}}$ and $p_{\text{2}}$ were shared hyperparameters, held constant and set independently of the user and task. Similar to the rationale behind input dose normalization, this choice enables fair comparison of learning rates across tasks by ensuring that differences in fitted parameters reflect true behavioral differences rather than scaling artifacts or task-dependent nonlinearities (at the cost of lower fitting performance, however, because the exponents were not individualized). Fixing these exponents across episodes also reduces model complexity, improves parameter identifiability, and reduces the risk of overfitting when estimating learning parameters from relatively sparse and noisy real-world behavioral data.

We incorporated process noise and measurement noise in the model to account for variability in real-world practice and sensor recordings. This choice is motivated by a growing body of evidence suggesting that multiple hidden processes govern motor learning, each with its own sources of uncertainty [18,28–30]. Specifically, process noise accounts for stochasticity in the evolution of internal memory states over time, due to fluctuations in attention, fatigue, motivation, or neural plasticity. Meanwhile, measurement noise captures variability in observed performance arising from sensor inaccuracies, inconsistent effort, or biomechanical variability in executing the same movement.

*Handling Temporal Sparsity and Set Breaks:* As noted above, real-world home rehabilitation training sessions with FitMi are not regularly spaced. Instead, sessions are typically distributed irregularly, often with multi-day gaps (“set breaks”) between episodes. To account for this sparsity, the state-space model applies updates only on observed training days. If two consecutive sessions occur on days $t_{n}$ and $t_{n+\text{1}}$, separated by $\Delta t=t_{n+\text{1}}-t_{n}$ days, the state update becomes:


$$\begin{array}{c} \begin{array}{c} X_{ik}(t+\text{1})=A_{ik}^{\Delta t}*X_{ik}(t)+A_{ik}^{\Delta t-\text{1}}*B_{ik}*{\left(U_{ik}(t)\right)}^{p_{\text{1}}}+{pn}_{ik}(t) \end{array} \end{array}$$ (3)

This formulation allows the internal memory state to decay passively over unobserved days, while attributing learning-related memory updates only to actual training inputs. It also avoids artificial imputation of behavior on missing days, maintaining biological and behavioral realism. During parameter estimation (see below), only observed data points are included in the expectation and maximization steps of the Expectation-Maximization (EM) algorithm [31].

#### The non-learning, performance-only, model.

To assess whether an episode reflects true motor learning or only transient performance changes, we defined an alternative, non-learning model. This reduced model excludes the memory state and assumes that observed performance is driven solely by the current input dose, i.e., practice on that day only:


$$\begin{array}{c} \begin{array}{c} Y_{ik}(t)=D_{ik}*{\left(U_{ik}(t)\right)}^{p_{\text{2}}}+{mn}_{ik}(t) \end{array} \end{array}$$ (4)

where the parameters $D_{ik}$, $p_{\text{2}}$, and ${mn}_{ik}(t)\;\sim \text{N}\left(\text{0},\;R_{ik}\right)$ are defined as in the learning model. This performance-only model serves as a null hypothesis: under this formulation, performance improvements reflect short-term responsiveness to input, without any retained learning.

### Parameter estimation via Expectation-Maximization (EM) algorithm

We estimated the parameters of the state-space model described above using the Expectation-Maximization (EM) algorithm, a probabilistic framework for maximum likelihood estimation in latent variable models. Although least-mean-square error (LMSE) fitting is widely used in motor learning research [26,32,33], it assumes that observed behavior reflects a deterministic function of learning and is agnostic to the stochasticity of the internal learning process itself. As a result, it can yield biased or overly confident parameter estimates, particularly when behavioral data are sparse, noisy, or irregular.

In contrast, explicitly modeling and estimating both output and process noise supports more realistic parameter estimation and improves the model’s ability to uncover latent learning processes from behavioral data. This approach has been shown to yield more robust inferences about motor learning in both human and animal studies [28,30,32]. EM is the algorithm of choice as it explicitly models both process noise and measurement noise, treating the hidden state trajectory $X_{ik}(t)$ as a latent variable [34–36]. This approach enhances the identifiability of learning dynamics from noisy, real-world performance data, particularly in settings such as home rehabilitation, where data collection is unsupervised and behavioral variability is high.

The EM algorithm iteratively alternates between inferring the posterior distribution over the latent state trajectory and updating model parameters to maximize the expected complete-data log-likelihood. In our implementation, following prior work in latent dynamical modeling of motor behavior [32], we used a Kalman smoother in the E-step to compute smoothed estimates of the latent trajectory, and combined closed-form updates and constrained optimization in the M-step to update parameters. Full algorithmic details are provided in S1 Text.

*Fixed Power Exponents and Model Selection.* The exponents $p_{\text{1}}$ and $p_{\text{2}}$ were treated as shared hyperparameters fixed across all doses and users (see above).

To identify appropriate values, we discretized the search space and performed a grid search over $\left(p_{\text{1}},\;p_{\text{2}}\right)\in {\left\{\text{0}.\text{1},\;\text{0}.\text{2},\;\ldots ,\;\text{1}.\text{0}\right\}}^{\text{2}}$. For each candidate pair, we estimated the other model parameters $\theta_{ik}$ using the EM algorithm and computed the log-likelihood across all episodes. The pair that yielded the highest overall likelihood was selected. The optimal $p_{\text{2}}$ value was then fixed and reused in the alternative (non-learning) model to ensure a consistent transformation of input dose for model comparison. For each episode, the learning model parameters were estimated as: $\theta_{\left(\text{1},\;ik\right)}=\left\{\tau_{ik},\;B_{ik},\;C_{ik},\;D_{ik},Q_{ik},R_{ik},\;X_{\text{1},\;ik},\;V_{\text{1},\;ik}\right\}$, while the non-learning model estimated a reduced set: $\theta_{\left(\text{0},\;ik\right)}=\left\{D_{ik},\;R_{ik}\right\}$. This setup enables a direct comparison between the full learning model and the simpler performance-only model, as detailed below.

*Initialization and Convergence Criteria*. To reduce sensitivity to local optima, initial parameters were selected using heuristics and coarse grid search on a representative subset of episodes. The EM algorithm was run until convergence, defined as a relative change in log-likelihood below a preset threshold (e.g., ${\text{10}}^{-\text{4}}$) or until reaching a maximum number of iterations (e.g., 200).

### Model comparison via likelihood ratio test (LRT)

To determine whether motor performance in each episode was better explained by a memory-based learning model or by a simpler, performance-only model, we conducted a model comparison using the likelihood ratio test (LRT). Using the log-likelihoods obtained from the EM algorithm for both the learning model ($\text{log}L_{\text{learn}}$) and the non-learning model ($\text{log}L_{\text{perf}}$), we computed the likelihood ratio statistic for each episode: $\Lambda =\text{2}\left(\text{log}L_{\text{learn}}-\text{log}L_{\text{perf}}\right)$. While the learning model, by design, fits the data better due to its greater flexibility, it also contains six more free parameters. The LRT accounts for model complexity by evaluating whether the improvement in log-likelihood is statistically justified, given the increase in parameter count.

Under standard regularity assumptions [37–39] the statistic Λ asymptotically follows a chi-squared distribution with degrees of freedom equal to the difference in the number of free parameters between the two models [39]. The LRT effectively tests the contribution of the additional six parameters unique to the learning model: $\tau_{ik},\;B_{ik},\;C_{ik},Q_{ik},\;X_{\text{1},\;ik},\;V_{\text{1},\;ik}$, and the test statistic follows a chi-squared distribution with 6 degrees of freedom.

Each episode was independently tested using this statistic. Episodes were classified as “learning” if the LRT yielded a statistically significant improvement in model fit (p<0.05). All remaining episodes were labeled as “non-learning.” This procedure enabled us to formally determine, for each (user, task) episode, whether including a latent memory mechanism significantly improved model fit beyond what could be explained by transient input–output effects alone.

We then conducted complementary analyses to evaluate the robustness of episode classification to multiple-testing correction and alternative model-selection criteria, and to characterize variation in the strength of evidence for learning across episodes. First, because episode length may influence the ability to distinguish between models, with shorter episodes providing less information for model comparison, we examined the distributions of likelihood ratio statistics and uncorrected p-values across all episodes. Second, we applied a Benjamini–Hochberg false discovery rate (FDR) correction across all episode-wise tests to account for multiple comparisons arising from testing episodes separately.

Finally, to quantify the relative support for the learning versus non-learning model on a continuous scale, we computed BIC-based model weights for each episode. The weight for the learning model was defined as ${\text{w}}_{\text{learn}}=\frac{\text{exp}\left(-\frac{\text{1}}{\text{2}}{\text{BIC}}_{\text{learn}}\right)}{\text{exp}\left(-\frac{\text{1}}{\text{2}}{\text{BIC}}_{\text{learn}}\right)+\text{exp}\left(-\frac{\text{1}}{\text{2}}{\text{BIC}}_{\text{perf}}\right)}$, with the weight for the non-learning model defined analogously, such that the two weights summed to 1 within each episode.

### Model validation via parameter recovery

Next, to evaluate the identifiability and robustness of parameter estimation in the presence of real-world variability and noise, we conducted a simulation-based validation procedure. This approach tests whether the model fitting procedure can reliably recover true parameter values when applied to synthetic data generated under realistic conditions.

For each episode in the dataset, we used the estimated parameters from the fitted learning model, along with the actual input sequence (daily normalized dose), to simulate synthetic output trajectories. Specifically, we generated simulated performance time series using the learning model equations, with added process and measurement noise drawn from the estimated noise distributions. In the simulations, we preserved the exact schedule of training (i.e., same days and doses) as in the original data.

To account for stochastic variability, we repeated this simulation process 100 times per episode, each with different random seeds for noise and initial conditions. For each simulated dataset, we re-estimated the model parameters using the EM algorithm, following the procedure described above. We then assessed parameter recovery by comparing the original (true) parameters with the re-estimated parameters across episodes, using the root mean square error (RMSE) and coefficient of determination ($R^{\text{2}}$). Strong recovery of key parameters, such as the retention time constant $\tau_{ik}$, learning rate $B_{ik}$, memory-performance gain $C_{ik}$, and input feedthrough $D_{ik}$, indicated that the model fitting procedure was robust to noise and variability and capable of recovering meaningful individual-specific learning dynamics.

## Results

### Outcome of data preprocessing

The final dataset comprised 4,661 episodes spanning 398 unique users and 20 distinct rehabilitation tasks. Each episode corresponds to a (user, task) pair, with behavioral data aligned and aggregated at the daily level following preprocessing.

S1 Fig summarizes the temporal structure and engagement patterns of these episodes. The distribution of episode durations, measured as the number of calendar days between the first and last training session in each episode (as a reminder, the data have been filtered to include episodes with at least 50 days for modeling purposes) is long-tailed (S1A Fig) with a median duration of 514 days. Note that some episodes lasted over 5 years, reflecting diverse real-world usage patterns. The number of training sessions per episode is also long-tailed, with a median of 89 sessions (S1B Fig). While most episodes contain fewer than 150 sessions, a subset of users engaged in intensive or high-frequency training. These findings highlight the heterogeneity of home rehabilitation behavior, both in the duration of user engagement and the frequency of training, supporting the need for individualized modeling.

**Fig 2:**
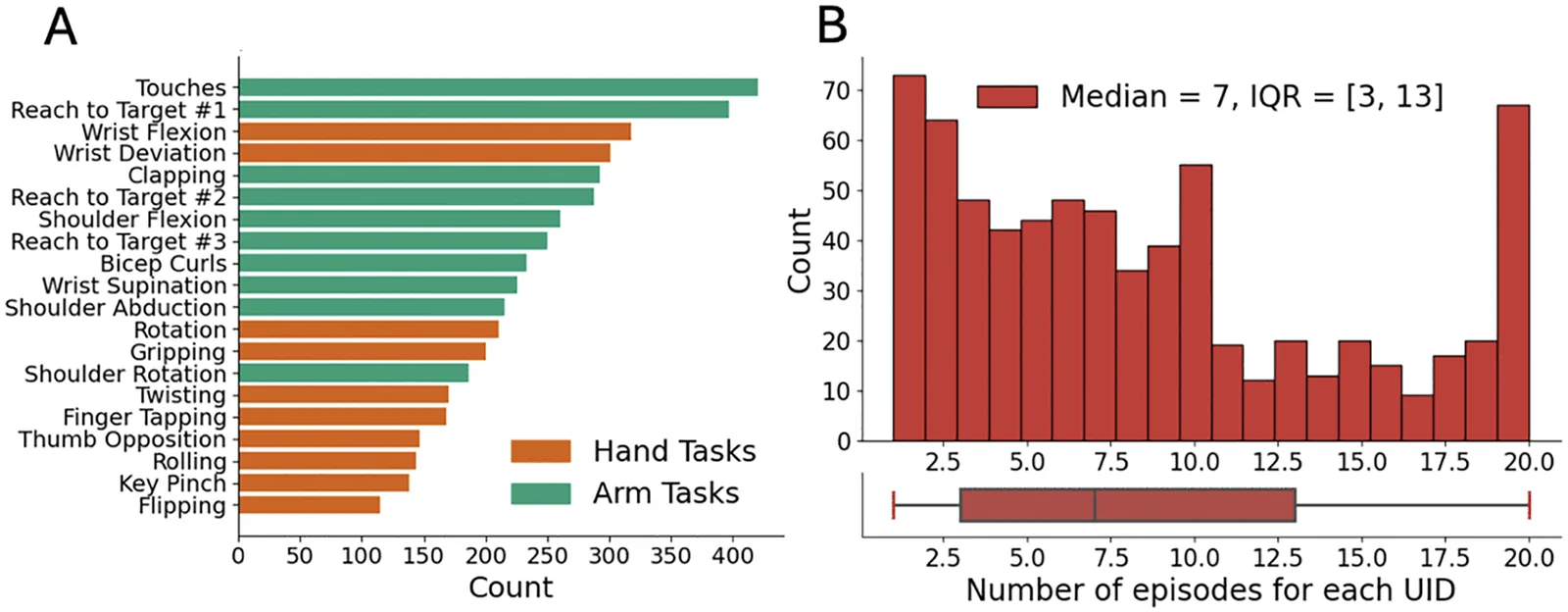
Distribution of episodes across tasks and users. A) Number of episodes per task, color-coded by task type (orange: hand tasks; green: arm tasks). Task popularity varied considerably, with some tasks (e.g., Touches, Reach to Target #1) being practiced over 400 episodes, while others were less frequently selected. B) Histogram and boxplot of the number of episodes per user (UID), where an episode is defined as engagement with the same task for at least 50 sessions.

To further describe the dataset structure, Fig 2A summarizes the distribution of the 4,661 episodes across tasks, revealing variation in the frequency with which different tasks were practiced. Fig 2B illustrates the distribution of episode counts per user, including the median and interquartile range (IQR) values. These results indicate that while many users trained on only one or two tasks, a subset practiced ten or more tasks, suggesting different usage styles (e.g., targeted versus comprehensive training). This variability in episode frequency across users and tasks informed our decision to fit models independently for each episode.

### Classification of learning and non-learning episodes

We applied the likelihood ratio test (LRT) to compare the full learning model against the reduced non-learning model for each of the 4,661 (user, task) episodes. Based on a significance threshold of p<0.05, 38.3% of episodes were classified as learning, and the remaining 61.7% as non-learning.

**Fig 3:**
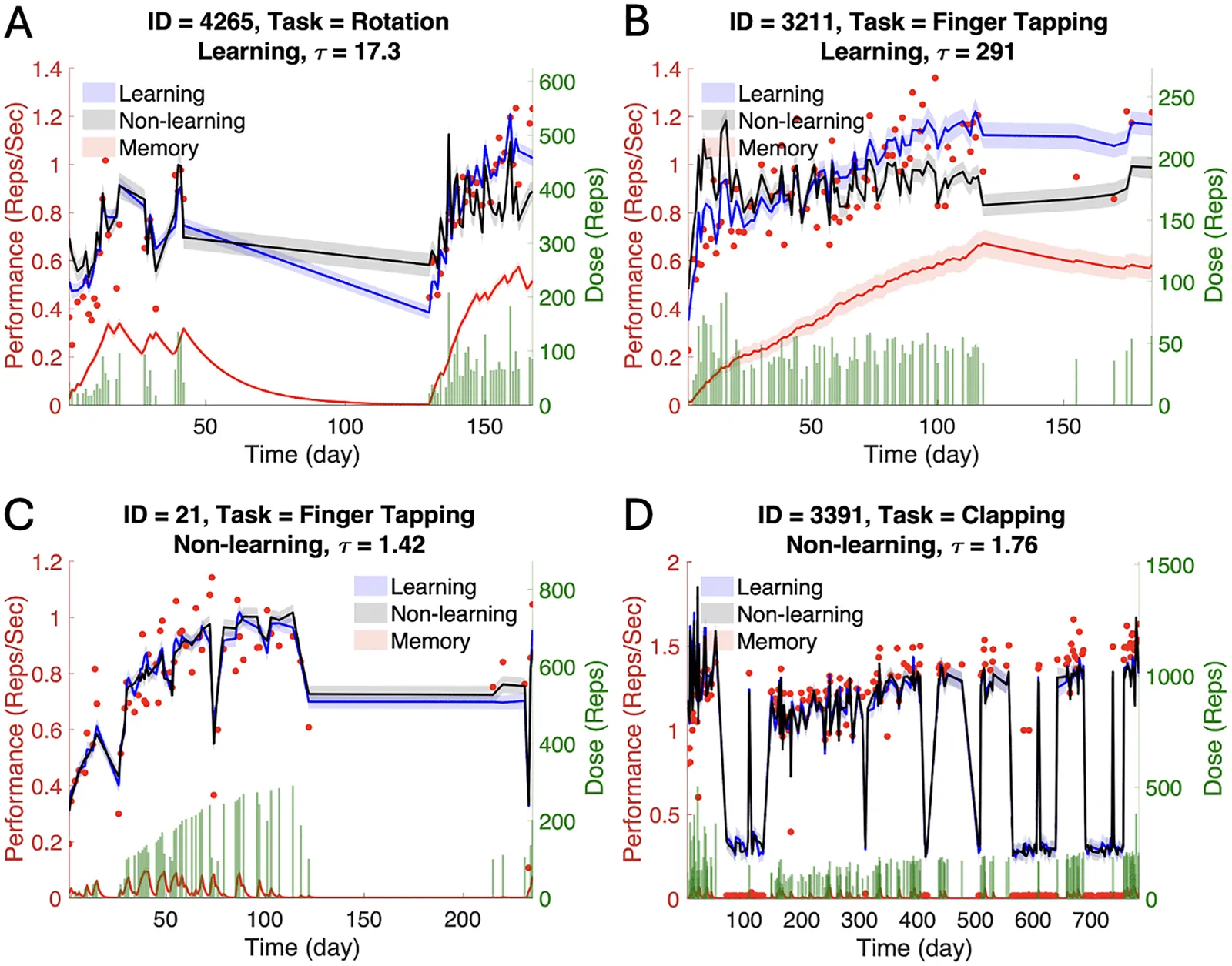
Examples of learning and non-learning episodes. Representative model fitting results for four (user, task) episodes illustrating both learning (top row) and non-learning (bottom row) classifications. Red dots represent observed performance (repetitions per second), green vertical bars indicate daily input dose (repetitions), and red curves show the inferred memory trajectories with 95% confidence bounds. The blue and gray curves represent the best-fitting learning and non-learning models, respectively. A) Example of a short-term learner (τ = 17.3). Performance improves with input but decays when training stops, indicating unstable memory. B) Example of a long-term learner (τ = 291). Performance remains stable despite sparse input, consistent with strong memory retention. C) Non-learning episode with no evidence of improvement (τ = 1.42). Performance fluctuates primarily as a function of the dose input on a given day. D) Non-learning episode flagged as a potential ceiling-effect case (τ = 1.76). Performance remains consistently high across many days (see text for details).

Fig 3 illustrates representative examples of each class. In each panel, the red-shaded line shows the inferred latent memory trajectory with 95% confidence bounds. The blue-shaded line represents the fitted output from the learning model, while the gray-shaded line shows the fit from the non-learning model. In learning episodes (top row), performance increases over time and is best explained by an accumulating memory state. In panel A, a short-term learner demonstrates performance that improves with input but decays when training stops, associated with a short decay time constant, τ, of 17.3 days. In contrast, in panel B, a long-term learner exhibits stable performance despite sparse input, characterized by a long decay time constant, τ, of 291 days and consistent memory retention. Conversely, non-learning episodes (bottom row) exhibit transient performance fluctuations, which are well captured by the input-driven non-learning model with little memory accumulation.

Following the primary episode-wise classification based on the likelihood ratio test (LRT), we conducted additional analyses to assess the robustness of this classification (S2 Text and S2 Fig). The distributions of likelihood ratio (LR) statistics (S2A Fig) and corresponding p-values (S2B Fig) showed a polarized pattern, with many episodes exhibiting either strong evidence for learning or little to no evidence of learning. After Benjamini–Hochberg FDR correction across 4,661 episodes (S2C Fig), only 27 (0.6%) changed classification. Consequently, 1,758 of the 1,785 episodes (98.5%) originally classified as learning at p < 0.05 remained significant. BIC-based learning weights (S2D Fig) showed a similarly polarized distribution, with only 98 episodes (2.1% of all 4,661 episodes) falling in the intermediate range between 0.1 and 0.9. Under BIC-based comparison, 144 episodes were reassigned as non-learning. This change represents 3.1% of all episodes and 8.1% of the 1,785 learning episodes, consistent with the stronger penalty for model complexity imposed by BIC. Overall, these complementary analyses indicate that the main episode-level classification was robust, while also showing that a small subset of episodes lay near the boundary between learning and non-learning.

We further evaluated model fit using the coefficient of determination ($R^{\text{2}}$) for both models in each episode (S3 Text and S3 Fig), which quantifies the proportion of variance in observed performance explained by each model’s predictions. Overall, the learning model showed a modestly higher mean fit than the non-learning model ($R^{\text{2}}$ = 0.616, 95% CI [0.612, 0.620] vs. $R^{\text{2}}$ = 0.579, 95% CI [0.575, 0.584]), corresponding to a significant paired difference ($\Delta R^{\text{2}}$ = 0.037, 95% CI [0.025, 0.048]; t = 11.75, p < 0.0001). When stratified by episode classification, learning episodes were substantially better explained by the learning model ($R^{\text{2}}$ = 0.642, 95% CI [0.635, 0.649] vs. $R^{\text{2}}$ = 0.548, 95% CI [0.540, 0.556]; $\Delta R^{\text{2}}$ = 0.083, 95% CI [0.070, 0.096]; t = 38.75, p < 0.0001), consistent with the presence of a memory-driven learning component. In contrast, non-learning episodes showed nearly identical fits under the two models ($R^{\text{2}}$ = 0.600, 95% CI [0.595, 0.605] vs. $R^{\text{2}}$ = 0.599, 95% CI [0.594, 0.604]; $\Delta R^{\text{2}}$ = 0.001), indicating that a simpler feedthrough-only model was sufficient. These results were consistent with the LRT-based classification: episodes classified as learning showed greater explanatory power under the learning model, whereas episodes classified as non-learning showed little meaningful difference between the two models.

### Ceiling effects in non-learning episodes

Not all episodes classified as non-learning reflect a lack of motor capacity. In some cases, performance may have reached an asymptotic plateau where additional practice yields no measurable improvement [40]. To identify these instances, we established a task-specific ceiling threshold based on the 95th percentile of performance across all episodes for each task. This threshold captures the empirically observed upper bound across participants while minimizing the influence of outliers.

We classified episodes as ceiling-effect cases if performance exceeded this 95th-percentile threshold in at least 20% of training sessions. This 20% operational cutoff, based on the distribution in S4 Fig, distinguishes sustained near-ceiling performance from isolated fluctuations. This approach is consistent with studies requiring effects to be observed across repeated measurements to ensure robust interpretation [41,42].

Using this definition, 279 of the 2,876 non-learning episodes (9.7%) were identified as ceiling-effect cases, while the remaining 2,597 (90.3%) were classified as true non-learning (S1 Table). As shown in Fig 3D, these episodes were often categorized as non-learning by the likelihood ratio test because a memory state did not significantly improve model fit at an already maximal functional level. In contrast, other non-learning episodes exhibited low or fluctuating performance that remained well below the ceiling, as seen in Fig 3C.

### Distributions of model parameters

To understand how parameter values differ between learning and non-learning episodes, we analyzed the estimated parameters of the learning model across all episodes. Although all episodes were fitted using the full learning model, we partitioned episodes by their LRT classification and examined the distributions of key parameters: the retention time constant $\tau_{ik}$, learning rate $B_{ik}$, memory-to-performance gain $C_{ik}$, and input feedthrough $D_{ik}$ in Eqs. (1) and (2).

**Fig 4:**
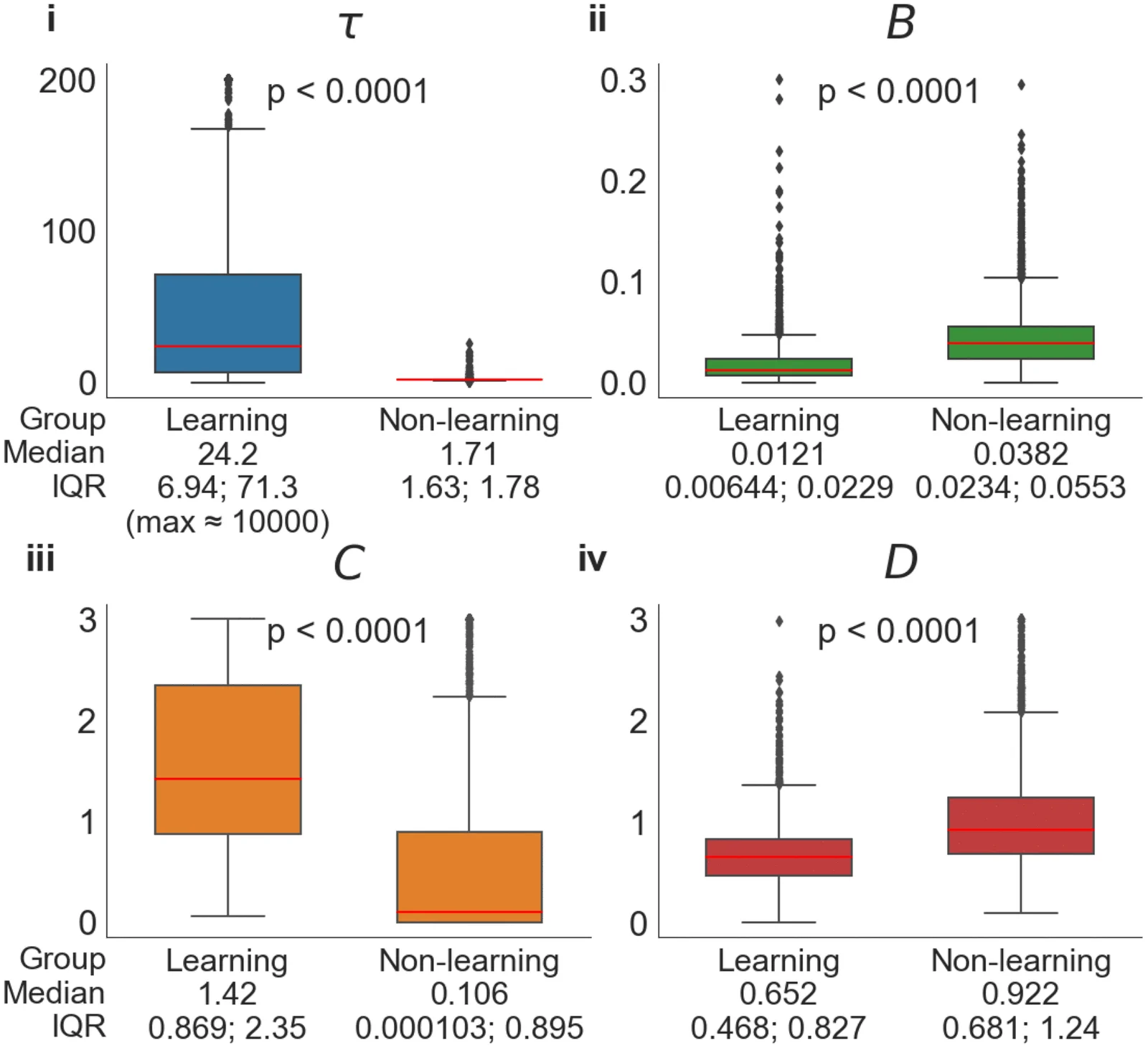
Distribution of state-space model parameters in learning vs. non-learning episodes. Estimated values of four key parameters are shown for episodes classified as “learning” (left group in each plot) and “non-learning” (right group), based on the likelihood ratio test. (A) Retention time constant τ, (B) Learning rate *B*, (C) Memory-performance gain *C*, and (D) Input feedthrough *D*- see Eqs. (1) and (2). Boxplots indicate the median (red line), interquartile range (IQR: box edges), whiskers (1.5 × IQR), and outliers (black points). Group medians and IQRs are printed below each plot. All group differences were statistically significant (Mann–Whitney U test, *p* < 0.0001).

As shown in Fig 4, there were clear differences between the two groups across multiple model parameters (Mann–Whitney U tests): i) The retention time constant τ was substantially higher in learning episodes (median = 24.2; IQR = [6.94, 71.3]) than in non-learning episodes (median = 1.71; IQR = [1.63, 1.78]), indicating longer-lasting memory effects (p < 0.0001). ii) Interestingly, the learning rate *B* tended to be smaller in learning episodes (median = 0.0121; IQR = [0.00644, 0.0229]) than in non-learning episodes (median = 0.0382; IQR = [0.0234, 0.0553]), possibly reflecting slower but more persistent accumulation of motor memory compared to transient bursts in non-learning cases (p < 0.0001). iii) The memory-performance gain *C* was also elevated in learning episodes (median = 1.42; IQR = [0.869, 2.35]) compared to non-learning episodes (median = 0.106; IQR = [0.000103, 0.895]), suggesting a stronger contribution of accumulated memory to observed performance (p < 0.0001). iv) In contrast, the input feedthrough parameter D was higher in non-learning episodes (median = 0.922; IQR = [0.681, 1.24]) than in learning episodes (median = 0.652; IQR = [0.468, 0.827]), consistent with immediate but short-lived effects of training dose (p < 0.0001).

Nonetheless, because the input feedthrough parameter D was greater than zero in learning episodes, performance in these episodes was due to both learning and the immediate effect of dose. Indeed, in the learning episodes, the median percentage of variance explained by learning was only 38.8%, with an interquartile range (IQR) of [19.2%, 68.7%] (S4 Text and S5 Fig). This variability across episodes suggests that while many learning episodes exhibit substantial memory-driven effects, others show more modest contributions, highlighting the heterogeneity of real-world rehabilitation behavior. These distribution patterns support the validity of the LRT-based classification and provide insight into the mechanisms underlying learning versus performance-driven behavior.

We next conducted two additional analyses that further support the adequacy of the modeling framework. First, a parameter correlation analysis indicated that overall parameter identifiability was acceptable, as most parameter pairs showed weak correlations across episodes (S5 Text and S6 Fig). The strong correlation between B and D (r = 0.857) in non-learning episodes is consistent with reduced identifiability of the learning-related component when τ is small. A moderate negative correlation between τ and B is also visible in both groups (r = -0.599 in learning episodes and r = -0.439 in non-learning episodes), reflecting the typical gain–time-constant tradeoff in first-order systems.

Second, we compared the full learning model against a series of simpler versions (S6 Text and S2 Table). Treating A, B, and the initial state as essential, we tested whether C, D, the process noise variance (Q), and the measurement noise variance (R) could be fixed at their group-mean estimates without degrading fit. Across all learning episodes, the full model consistently performed best, yielding the lowest mean BIC (−209.1) and the highest mean log-likelihood (124.2). While simpler models fixing only Q or C showed similar performance, none outperformed the full model. Models with multiple fixed parameters showed progressively poorer fit, supporting the retention of all eight free parameters for this dataset.

### Long-term vs. short-term learners

As shown in Fig 3B, where performance is maintained despite sparse training between days 120 and 170, a portion of learning episodes exhibited strong memory retention with large time constants. In these cases, the retention factor A approached 1, indicating minimal decay in the latent state *X* without further input. To distinguish between long-term and short-term learners, we set an operational threshold of τ = 30 days. Among learning episodes, 44.6% met this criterion, consistent with durable motor memory.

This 30-day threshold provides a practical boundary between persistence measured in weeks versus months, allowing for a clear distinction between short-lived and durable effects in home-based rehabilitation [8,9,27,43]. While shorter time constants were more frequent (S7A Fig), the proportion of long-term episodes decreased smoothly as the threshold increased, with no natural breakpoint (S7B Fig).

### Task- and user-level learning classification

**Fig 5:**
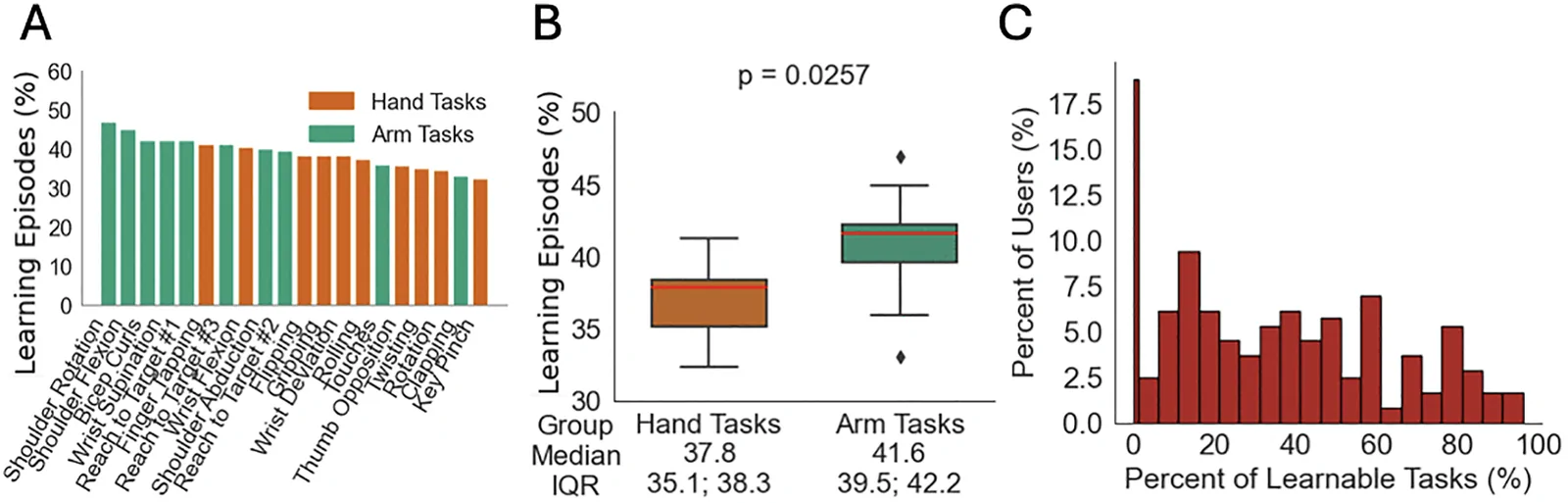
Task-level and user-level learning classification. A) Percentage of episodes classified as learning for each of the 20 upper-limb exercises. B) Boxplot comparing the percentage of episodes classified as learning between hand tasks (left) and arm tasks (right). Group medians and IQRs are shown below the plot. C) Histogram of user-level learning profiles, based on users who practiced at least five different tasks. For each user, we calculated the percentage of tasks they learned.

To investigate how motor learning varies, we analyzed classification distributions at both the task and user levels. Fig 5A shows the percentage of learning episodes for each of the 20 upper-limb exercises, aggregated across users and sorted by frequency. The results reveal a continuous distribution of task learnability rather than a dichotomy between learnable and non-learnable tasks. This gradual variation suggests that learning outcomes depend not solely on task difficulty but also on user-specific factors such as ability, engagement, or impairment level. However, most hand tasks exhibited fewer learning episodes than arm tasks (Fig 5A). We therefore compared the percentage of learning episodes between hand and arm tasks using a Mann–Whitney U test. Fig 5B shows a larger percentage of learnable arm tasks than hand tasks (p = 0.0257).

Fig 5C summarizes user-level learning behavior. Among users who practiced at least five different tasks, we computed the percentage of tasks for which they were classified as learners. The histogram shows that most users exhibited a mixture of learning and non-learning across tasks. Only a small minority, approximately 5%, learned all tasks practiced, while 18% were classified as non-learners with no detected learning. The remaining 77% demonstrated selective learning, underscoring the individualized and task-dependent nature of motor skill acquisition in unsupervised rehabilitation.

In the FitMi home program, users can practice any task they choose. Thus, to examine whether the classification of non-learners could be influenced by task selection bias, we compared the task distributions of users with zero learning episodes and users with a high proportion of learning episodes (S7 Text and S8 Fig; note that the high-learning group was defined as users with ≥ 71% learning episodes to yield a group size similar to that of the zero-learning group). Zero-learning users engaged in a higher proportion of arm-related tasks (65.6%) than high-learning users (57.6%), even though arm-related tasks were, on average, more learnable than hand-related tasks in our analysis. We further quantified the difference in task composition between the two groups using the total variation distance (TVD), which measures the overall dissimilarity between two distributions. The observed difference was modest (TVD = 0.141), indicating limited divergence in task composition between groups, and was not statistically significant in a permutation test (p = 0.082). These results provide little evidence that the non-learner classification was primarily driven by task selection bias.

### Training patterns in learners vs. non-learners

**Fig 6:**
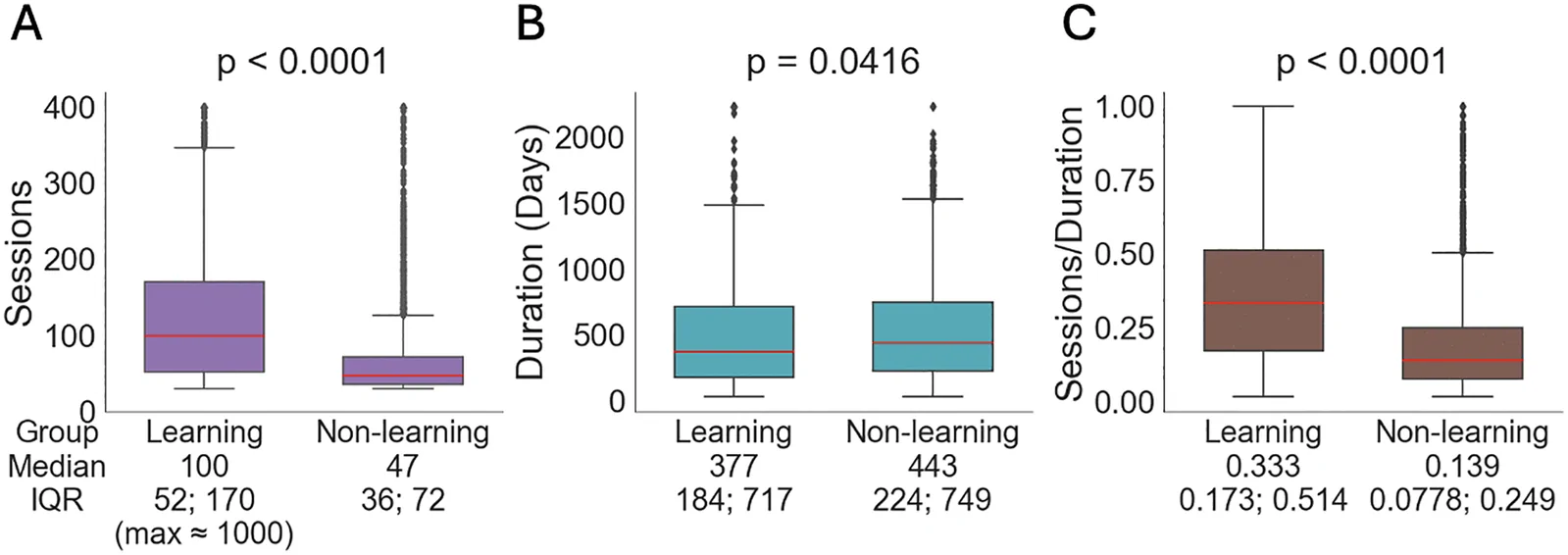
Comparison of training schedule statistics between learning and non-learning episodes. Differences between “learning” (left group in each plot) and “non-learning” (right group) episodes across three measures of training behavior: (A) Total number of sessions completed, (B) Duration of training in days (from the first to the last recorded session), and (C) Session frequency (sessions per day). Boxplots indicate the median (red line), interquartile range (IQR: box edges), whiskers (1.5 × IQR), and outliers (black points). Group medians and IQRs are printed below each plot. All group differences were statistically significant (Mann–Whitney U test), with p < 0.0001 for sessions and frequency, and p = 0.0416 for duration.

To examine how training behavior relates to learning outcomes, we compared the distributions of episode duration, number of sessions, and session frequency between episodes classified as learners and non-learners (Fig 6). The episode duration was defined as the number of days from the first to the last session. The number of sessions captured how many distinct practice days occurred within the episode. The session frequency was computed as the ratio of sessions to duration (sessions per day).

Fig 6 shows that clear differences also emerged between learning and non-learning episodes in terms of training behavior and schedule regularity: i) There were substantially greater number of sessions in learning episodes (median = 100; IQR = [52, 170]) than in non-learning episodes (median = 47; IQR = [36, 72]; p < 0.0001) indicating more repeated engagement. ii) Surprisingly, despite comprising fewer sessions, non-learning episodes lasted longer (median = 443 days; IQR = [224, 749]) than learning episodes (median = 377 days; IQR = [184, 717]; p = 0.0416), suggesting that learners condensed their training into fewer days. iii) As a result, learning episodes exhibited higher session frequency, quantified by sessions per day (median = 0.333; IQR = [0.173, 0.514]), compared to non-learning episodes (median = 0.139; IQR = [0.0778, 0.249], p < 0.0001), reflecting more consistent and intensive practice.

### Parameter recovery via simulations

As shown in S9 Fig, a simulation-based parameter recovery analysis provided strong evidence that the parameters inferred from real-world FitMi data are not artifacts of overfitting or noise but rather reflect recoverable, interpretable motor learning characteristics.

To quantitatively assess recovery performance, we computed the root mean square error (RMSE) and coefficient of determination ($R^{\text{2}}$) between true and estimated parameters across all simulated episodes. These metrics confirmed high recovery accuracy for *B* (RMSE = 0.0144, $R^{\text{2}}=\text{0}.\text{93}\text{2}$), *C* (RMSE = 0.482, $R^{\text{2}}=\text{0}.\text{911}$), and *D* (RMSE = 0.206, $R^{\text{2}}=\text{0}.\text{928}$), indicating low estimation error and strong correspondence with ground truth. For τ, RMSE was higher (RMSE = 14.4, $R^{\text{2}}=\text{0}.\text{903}$) compared to other parameters, reflecting increased sensitivity at larger values. However, the high $R^{\text{2}}$ indicates that the relative ordering of τ values was well preserved despite increased absolute error. Additional analyses further showed that the absolute error in τ estimation increased as the true τ increased, whereas the relative error remained stable, and recovery improved with a greater number of training sessions (S10 Fig). Together, these findings indicate that the model can reliably recover key parameters, including the forgetting time constant, particularly when more observations provide stronger constraints for parameter estimation.

## Discussion

This study presents a scalable, model-based framework for assessing motor learning in unsupervised, at-home rehabilitation. By fitting a latent state-space model to 4,661 episodes and using a likelihood ratio test to account for model complexity, we identified episodes reflecting memory-based learning dynamics, as distinct from transient performance fluctuations. The model incorporates process and measurement noise to capture day-to-day variability, with an EM algorithm providing robust estimation of latent parameters even under irregular practice schedules.

Our results demonstrate that learning is detectable even in highly variable, real-world data, as 38.3% of episodes were classified as learning using a likelihood ratio test. However, while long-term motor plasticity is often emphasized in rehabilitation [44,45], only 44.6% of learning episodes, corresponding to 17.1% of all episodes, exhibited retention time constants longer than 30 days, indicating that most learning was relatively short-term. Note that the 30-day threshold was chosen because it provides a clear distinction between short-lived and durable effects sensorimotor rehabilitation. Nonetheless, as shown in additional analyses (S7A and S7B Fig), it is better viewed as a clinically meaningful and practical benchmark rather than a unique natural boundary in the data.

Among the 61.7% of non-learning episodes, some may reflect ceiling effects. Using a task-specific threshold (95th percentile of performance), we identified that 9.7% of these episodes maintained near-maximal performance in at least 20% of sessions. The remaining 90.3% of non-learning episodes (55.7% of all episodes, see S1 Table) likely represent genuine non-learning, potentially due to low engagement, suboptimal task selection, or severe impairment. However, non-learning episodes are not necessarily treatment failures. In stroke rehabilitation, maintaining function over extended periods is clinically meaningful, especially for those at risk of decline. These episodes may reflect performance plateaus, the maintenance of previously acquired skills, or compensatory strategies that preserve stability without producing measurable gains in repetition rate.

Identifying ceiling-effect episodes improved the interpretability of the non-learning category by distinguishing performance saturation from a genuine lack of learning. A sensitivity analysis showed that the estimated proportion of ceiling-effect episodes declined steeply at low session cutoffs but changed more gradually beyond approximately 20%, indicating that classification is highly sensitive to threshold choice at low values but stabilizes thereafter. Nevertheless, the identification of ceiling-effect episodes remains based on somewhat arbitrary criteria. Future studies could determine whether these episodes truly reflect ceiling effects, that is, whether the skill has become sufficiently automatized to require minimal cognitive resources, by incorporating a secondary cognitive task during training [42,46]. If primary-task performance remains maximal while secondary-task performance declines, this would suggest that the primary task still requires cognitive resources despite asymptotic performance, indicating that the apparent ceiling may reflect limited sensitivity of the performance measure rather than true behavioral automaticity.

A key strength of our approach is its ability to generate personalized, per-episode estimates of learning across a diverse set of tasks and users. We observed a wide range of learners: some users learned across all tasks, others showed selective learning, and some exhibited no detected learning at all. Similarly, some tasks were learnable for many users, while others showed mixed outcomes. These observations suggest that neither users nor tasks fit neatly into binary “learnable” or “non-learnable” categories, but rather that learning lies on a continuum shaped by user-level factors (e.g., neurological status, motivation) and task-level factors (e.g., difficulty, biomechanical demands), as well as their interactions [12,40,47]. Relatedly, and although the distinction between “learning” and “non-learning” episodes is useful conceptually even at the episode level, learning exists on a continuum rather than as a strict binary behavior. This is shown by fitting the full model to non-learning episodes, which revealed small but positive median time constants (median 1.71 days IQR = [IQR: 1.63; 1.78]; in addition, the learning rate in non-learning episodes was also non-zero (median = 0.0382; IQR = [0.0234, 0.0553]), and interestingly greater than that observed for learning episodes (median = 0.0121; IQR = [0.00644, 0.0229]). Therefore, non-learning episodes show some evidence of fast-learning, fast-forgetting behavior. In future work, models with multiple states will be used to better characterize this learning continuum (see Limitations and future work section below).

Furthermore, arm tasks were found to be more learnable than hand tasks. At least two explanations can be considered. First, this may be due to the FitMi sensors being more sensitive to large arm movements than subtle finger control. Second, hand recovery is often slower and more limited because it relies heavily on the corticospinal tract, which is frequently damaged, whereas gross arm movements can utilize alternative neural pathways [48,49].

Learning episodes were associated with higher training frequency over shorter durations, while non-learning episodes involved less frequent training spread across longer spans. However, this relationship does not establish causality. A concentrated schedule may facilitate learning, but participants who were more capable or motivated might simply have trained more often. Furthermore, unmeasured confounders, such as baseline impairment, caregiver support, and task difficulty, likely shaped both training behavior and outcomes. Prospective studies are required to determine whether training structure directly influences motor learning.

### Limitations and future work

Although our proposed framework successfully quantifies learning dynamics in unsupervised settings, several limitations must be addressed before it can be translated into predictive clinical tools. First, the dataset did not include subject-level covariates (e.g., age, impairment severity, chronicity, or cognitive status) which prevented us from linking parameters such as learning rates to clinical factors. Consequently, heterogeneity may reflect baseline ability rather than intrinsic learning capacity. This limits the framework to retrospective analysis; it can identify unproductive tasks after the fact but cannot yet predict which new tasks will be most beneficial for a specific patient. Therefore, future iterations should incorporate patient-level data to update predictions as new data arrive. This would support early model-based identification of task responsiveness and enable proactive, personalized precision rehabilitation [50–53].

Second, our definition of learning is inherently model-based, relying on likelihood ratio tests rather than external validation. While statistically principled, this approach infers the latent memory state solely from performance time series, lacking independent behavioral or physiological markers. For instance, contextual factors such as fatigue, motivation, or task switching may be absorbed into the estimated decay rate, potentially biasing the interpretation of retention as a purely memory-driven process. To address this, model-derived classifications should be validated against independent behavioral or neurophysiological markers in future research.

Third, we used a parsimonious structure (single latent state, fixed exponents) to fit sparse data, which likely oversimplifies recovery dynamics. A single state compresses processes across distinct timescales, such as fast adaptation and long-term consolidation [26,54,55], and cannot separate compensation from true recovery. In addition, while the fixed exponents $\left(p_{\text{1}},\;p_{\text{2}}\right)\;$enable standardized comparison of learning rates *B* and feedthrough *D*, they may reduce flexibility for capturing individualized learning dynamics because they assume that the sublinear dose-response relationship reflecting diminishing efficiency is the same across all episodes and learners. Finally, the model ignores cross-task interactions such as transfer or interference [21], as well as the hierarchical structure of the dataset, where observations are nested within users and tasks, all of which can influence estimates of learning and retention. To account for these complexities, more flexible structures, including multi-state models and hierarchical Bayesian approaches (e.g., [22]), should be used in future research to capture learning across distinct timescales and tasks. For instance, it is possible that the non-linear dose-response curve for learning (as parametrized by $p_{\text{1}}$) is subject- and/or task-specific, and future task- and subject- hierarchical models could capture these relationships.

Fourth, repetitions per second served as a performance proxy because the system only records contact times. In rehabilitation, higher repetition rates generally reflect improved movement fluency and reduced execution time [13,56]. While this metric is a valid indicator of motor performance that accounts for approximately 70% of the variance in Upper Extremity Fugl-Meyer scores in people post-stroke [57], it is sensitive to effort, motivation, or fatigue. Furthermore, without joint kinematics, we cannot distinguish neurological restitution from compensatory strategies, such as trunk leaning [58,59]. This is critical as participants may improve in task space without recovering the joint space patterns of healthy controls [60]. To distinguish restitution from compensation, richer sensing modalities, such as video-based motion capture, should be integrated to enable 3D joint-level analysis and a more granular assessment of movement quality [61,62].

Finally, the model provides limited guidance for patients who show no detectable learning across tasks. While task-level analysis can help clinicians identify unproductive exercises, non-learning should not be dismissed as failure. In many settings, therapy continues based on medical necessity [16] because maintaining function is vital to prevent deterioration. Because arm function and use may reinforce each other [16,63], preserving stable performance is clinically valuable and can counteract the often-observed decay of therapy gains. Future research should therefore focus on expanding the framework to quantify functional maintenance and stability as meaningful clinical outcomes alongside active learning.

By automating the assessment of motor learning, our study lays the foundation of data-driven and personalized rehabilitation technologies. Future studies should directly link model-derived learning metrics to independent clinical measures and evaluate whether they can improve individualized task selection. Establishing this empirical link through controlled trials will be essential to translate these computational insights into actionable clinical protocols.

## Supporting information

S1 Text Expectation-Maximization (EM) algorithm for state-space model estimation. (DOCX)

S2 Text Robustness of learning classification. (DOCX)

S3 Text$R^{2}$ for learning vs. non-learning episodes.(DOCX)

S4 Text Percent of performance variance explained by learning for learning episodes. (DOCX)

S5 Text Parameter correlation analysis for learning vs. non-learning episodes. (DOCX)

S6 Text Model comparison between learning model vs. simpler models. (DOCX)

S7 Text Task distribution for users with zero learning and high learning. (DOCX)

S1 Fig Distributions of episode duration and training sessions. (DOCX)

S2 Fig Robustness of learning classification. (DOCX)

S3 Fig$R^{2}$ for learning vs. non-learning episodes.(DOCX)

S4 Fig Percentage of ceiling effect for non-learning episodes. (DOCX)

S5 Fig Percent of performance variance explained by learning for learning episodes. (DOCX)

S6 Fig Parameter correlation analysis for learning vs. non-learning episodes. (DOCX)

S7 Fig Percentage of long-term learning for learning episodes. (DOCX)

S8 Fig Task distribution for users with zero learning and high learning. (DOCX)

S9 Fig Parameter recovery via simulation. (DOCX)

S10 Fig Recovery error for forgetting time constant. (DOCX)

S1 Table Percentage of ceiling effect for non-learning episodes. (DOCX)

S2 Table Model comparison between learning model vs. simpler models. (DOCX)
