# Supplementary material for: To learn or not to learn? Model-based estimation of motor learning in a large, “in the wild”, home rehabilitation database
Source: PLOS Digit Health. 2026 Aug 27;5(8):e0001598. doi: 10.1371/journal.pdig.0001598 (PMC13521380; doi:10.1371/journal.pdig.0001598)
Supplement: Supplementary file 1 [file pdig.0001598.s001.docx]

**S1. Expectation-Maximization (EM) algorithm for state-space model estimation**

To estimate parameters of the first-order, discrete-time stochastic state-space model described in the main text, we used the Expectation-Maximization (EM) algorithm—a probabilistic framework for maximum likelihood estimation in latent variable models [31, 63, 64].

Let $\left\{ X_{ik}\left( t \right) \right\}_{t=1}^{T}$ denote the latent memory trajectory for episode $\left( i, k \right)$, and let $\theta$ denote the set of model parameters. EM alternates between two steps:

**S1.1. E-step: expectation via Kalman smoothing**

In the E-step, we estimate the posterior distribution over the latent state trajectory given the observed data and current parameter estimates $\theta^{\left( n \right)}$. This is achieved via a two-pass procedure:

- *Forward pass (Kalman filtering)*: At each time step $t$, the Kalman filter computes the filtered estimate of the latent state $X_{ik}\left( t \right)$ and its uncertainty, using all observations up to $t$. These estimates are optimal under linear-Gaussian assumptions but do not incorporate future information.
- *Backward pass (Rauch–Tung–Striebel (RTS) smoothing)*: Starting at the final time step, the RTS smoother propagates information backward in time to refine earlier state estimates based on the full observation sequence. This yields smoothed expectations of the latent trajectory $\left\{ X_{ik}\left( t \right) \right\}_{t=1}^{T}$, which are then used to compute the expected complete-data log-likelihood.

**S1.2. M-step: maximization of the expected log-likelihood**

In the M-step, we maximize the expected complete-data log-likelihood with respect to the model parameters: $\theta=\left\{ \tau_{ik}, B_{ik}, C_{ik}, D_{ik},Q_{ik},R_{ik}, X_{1, ik}, V_{1, ik} \right\}$. This model includes eight free parameters per episode. Updates were performed as follows:

- *Closed-form updates* were used for the process noise covariance $Q_{ik}$, measurement noise covariance $R_{ik}$, initial state mean $X_{1, ik}$, and initial variance $V_{1, ik}$, following standard Kalman EM formulations.
- *Training sensitivity parameters* $B_{ik}, C_{ik}, D_{ik}$ were updated via regularized least-squares regression on the smoothed latent states and inputs.

The retention parameter $\tau_{ik}$ was constrained to be strictly positive ($\tau_{ik}>0$) and optimized numerically. We implemented retention via an exponential decay term $A_{ik}=e^{-\frac{1}{\tau_{ik}}}$, ensuring the interpretability of $\tau_{ik}$ as a time constant.

The E and M steps were iterated until convergence, defined as either a relative change in log-likelihood below ${10}^{-6}$ or a maximum of 200 iterations.
