# Supplementary material for: To learn or not to learn? Model-based estimation of motor learning in a large, “in the wild”, home rehabilitation database
Source: PLOS Digit Health. 2026 Aug 27;5(8):e0001598. doi: 10.1371/journal.pdig.0001598 (PMC13521380; doi:10.1371/journal.pdig.0001598)
Supplement: Supplementary file 2 [file pdig.0001598.s002.docx]

**S2. Robustness of learning classification**

To further evaluate the robustness of the episode-level learning classification, we examined the distributions of likelihood ratio (LR) statistics, uncorrected p-values, FDR-adjusted p-values, and BIC-based learning weights across all 4,661 episodes (S2 Fig).

As shown in S2 Fig A, the distribution of LR statistics was highly right skewed, with a pronounced peak near zero and a long right tail. This pattern indicates that for many episodes, the full learning model provided little improvement in fit to the observed data over the non-learning model, whereas a subset of episodes showed strong evidence in favor of the learning model. Consistent with this, the distribution of uncorrected p-values was bimodal, with many episodes clustered near 0 or 1 (S2 Fig B). Episodes with p-values near 0 indicate strong evidence for learning, whereas those with p-values near 1 indicate little to no evidence for learning. The distribution of FDR-adjusted p-values showed a very similar pattern (S2 Fig C), indicating that the main episode-level classification was highly robust to correction for multiple comparisons. Only 27 episodes changed classification from learning to non-learning after FDR correction, corresponding to 0.58% of all 4,661 episodes and 1.51% of the 1,785 episodes originally classified as learning.

The distribution of BIC-based weights for the learning model was also strongly polarized, with many episodes clustering near 0 or 1 (S2 Fig D), indicating strong support for the non-learning or learning model, respectively. At the same time, the BIC weights provided a continuous measure of evidence for learning and therefore identified intermediate cases that were not fully captured by binary classification. However, such cases were relatively uncommon: only 98 of 4,661 episodes (2.10%) had BIC-based learning weights between 0.1 and 0.9. Under BIC-based model comparison, 144 episodes were reassigned from the learning to the non-learning category, corresponding to 3.09% of all 4,661 episodes and 8.07% of the 1,785 episodes originally classified as learning. Although this proportion was larger than that observed under FDR correction, the overall pattern remained broadly consistent across methods. This difference is consistent with the stronger penalty for model complexity imposed by BIC, which makes model selection more conservative for borderline episodes.
