# Supplementary material for: To learn or not to learn? Model-based estimation of motor learning in a large, “in the wild”, home rehabilitation database
Source: PLOS Digit Health. 2026 Aug 27;5(8):e0001598. doi: 10.1371/journal.pdig.0001598 (PMC13521380; doi:10.1371/journal.pdig.0001598)
Supplement: Supplementary file 3 [file pdig.0001598.s003.docx]

**S3.** $\boldsymbol{R}^{\mathbf{2}}$ **for learning vs. non-learning episodes**

To assess the model fit quality for each training episode, we computed the coefficient of determination ($R^{2}$) separately for both the learning model (state-space model with memory term) and the non-learning model (feedthrough-only model). For each episode, $R^{2}$ was calculated as:

$\begin{aligned} R^{2}=1-\frac{\sum_{t} \left( Y\left( t \right)-\hat{Y}\left( t \right) \right)^{2}}{\sum_{t} \left( Y\left( t \right)-\bar{Y} \right)^{2}}\#\left( 5 \right) \end{aligned}$

where $Y\left( t \right)$ is the observed performance, $\hat{Y}\left( t \right)$ is the model prediction, and $\bar{Y}$ is the mean of the observed values. Episodes were classified as “learning” or “non-learning” based on the likelihood ratio test between the two models. We then plotted the $R^{2}$ values for each episode as a scatter plot (S3 Fig), with the non-learning model $R^{2}$ on the $x$-axis and the learning model $R^{2}$ on the $y$-axis. The diagonal line $y=x$ indicates equal fit quality between models.

The results reveal distinct patterns. Overall, the learning model achieved a higher mean fit than the non-learning model ($R^{2}$ = 0.616, 95% CI [0.612, 0.620] vs. $R^{2}$ = 0.579, 95% CI [0.575, 0.584]), corresponding to a significant paired difference ($\Delta R^{2}$ = 0.037, 95% CI [0.025, 0.048]; t = 11.75, p < 0.0001). When stratified by episode classification:

- For **learning episodes**, the learning model showed a notably better fit than the non-learning model ($R^{2}$ = 0.642, 95% CI [0.635, 0.649] vs. $R^{2}$ = 0.548, 95% CI [0.540, 0.556]; $\Delta R^{2}$ = 0.083, 95% CI [0.070, 0.096]; t = 38.75, p < 0.0001), supporting the presence of memory-driven adaptation.
- For **non-learning episodes**, the $R^{2}$ values were similar across models ($R^{2}=0.600, 95\% CI [0.595, 0.605] vs. R^{2}=0.599, 95\% CI [0.594, 0.604]; \Delta R^{2}=0.001, 95\% CI [-0.006, 0.008]; t=4.27, p<0.0001$), suggesting that a simpler, feedthrough-only model was sufficient to capture the behavior.

These findings support the model selection criterion: the learning model is more appropriate when memory-dependent adaptation occurs, while the non-learning model suffices in its absence.
