# Supplementary material for: To learn or not to learn? Model-based estimation of motor learning in a large, “in the wild”, home rehabilitation database
Source: PLOS Digit Health. 2026 Aug 27;5(8):e0001598. doi: 10.1371/journal.pdig.0001598 (PMC13521380; doi:10.1371/journal.pdig.0001598)
Supplement: Supplementary file 4 [file pdig.0001598.s004.docx]

**S4. Percent of performance variance explained by learning for learning episodes**

To quantify the contribution of memory-driven learning to performance improvement in each episode classified as a “learning” episode, we computed the percentage of output variance explained by the internal memory state. According to the learning model equation:

$$\begin{aligned} Y_{ik}\left( t \right)=C_{ik}*X_{ik}\left( t \right)+D_{ik}*\left( U_{ik}\left( t \right) \right)^{p_{2}}+{mn}_{ik}\left( t \right)\#\left( 6 \right) \end{aligned}$$

We isolated the contribution of the memory-dependent term, $C_{ik}*X_{ik}$, and computed the percentage of variance in the observed output $Y_{ik}$ explained by this component as:

$$\begin{aligned} Percent Explained by Learning (\%) =\frac{\mathrm{Var}\left( C_{ik}*X_{ik} \right)}{\mathrm{Var}\left( Y_{ik} \right)}\times100\#\left( 7 \right) \end{aligned}$$

This metric captures the degree to which memory, as accumulated over time through training, accounts for performance variation. A higher value suggests stronger memory-driven learning, whereas a lower value indicates greater influence from feedthrough input or motor noise.

S5 Fig shows the distribution of this percentage across all learning episodes. The histogram and accompanying boxplot reveal a wide range of learning contributions.
