# Supplementary material for: To learn or not to learn? Model-based estimation of motor learning in a large, “in the wild”, home rehabilitation database
Source: PLOS Digit Health. 2026 Aug 27;5(8):e0001598. doi: 10.1371/journal.pdig.0001598 (PMC13521380; doi:10.1371/journal.pdig.0001598)
Supplement: Supplementary file 5 [file pdig.0001598.s005.docx]

**S5. Parameter correlation analysis for learning vs. non-learning episodes**

Because parameter separability may be problematic in real-world data with sparse observations and irregular sampling intervals, we conducted parameter correlation analysis for the recovered parameter estimates to assess practical identifiability.

S6 Fig shows correlation matrices for (A) learning episodes and (B) non-learning episodes. Overall, most parameter pairs showed weak correlations, indicating that the model parameters were generally well identifiable.

In the learning episodes, no strong correlations were observed, suggesting that the parameters could typically be estimated independently. In contrast, in the non-learning episodes, a strong positive correlation was observed between B and D ($r=0.857$). This pattern reflects a parameter identifiability issue specific to the non-learning regime rather than a meaningful coupling between distinct effects. Specifically, because $\tau$ is typically small in non-learning episodes, the learning-related component becomes effectively instantaneous, making B and D exert approximately the same direct effect on performance. Under these conditions, the two parameters are difficult to distinguish statistically and therefore appear highly correlated. Importantly, this identifiability issue does not affect the main analysis, because the learning component is removed for episodes classified as non-learning.

We also note a moderate negative correlation between $\tau$ and B ($r=-0.599$ in learning episodes and $r=-0.439$ in non-learning episodes). This pattern is consistent with the well-known gain–time-constant tradeoff in first-order systems, whereby changes in one parameter can partially compensate for changes in the other during model fitting. **Accordingly, estimates of** $\tau$ **are expected to be somewhat less precise than those of the other parameters, consistent with the parameter recovery results shown in** S9 Fig. As further illustrated in S10 Fig C, the estimation error for the time constant of decay decreases as the number of training sessions increases, indicating that additional data provide stronger constraints on $\tau$.

Taken together, these findings suggest that overall parameter identifiability was acceptable, although the time constant was estimated less precisely than the other parameters, particularly in sparsely sampled episodes.
