# Supplementary material for: To learn or not to learn? Model-based estimation of motor learning in a large, “in the wild”, home rehabilitation database
Source: PLOS Digit Health. 2026 Aug 27;5(8):e0001598. doi: 10.1371/journal.pdig.0001598 (PMC13521380; doi:10.1371/journal.pdig.0001598)
Supplement: Supplementary file 6 [file pdig.0001598.s006.docx]

**S6.** **Model comparison between learning model vs. simpler models**

Although the EM algorithm combined with Kalman smoothing can, in principle, identify all eight free parameters, parameter separability may be more challenging in real-world data with sparse observations and irregular sampling intervals. **To evaluate whether a simplified parameterization could provide an equally adequate description of the data, we compared the learning model with a series of simpler models fit to episodes classified as learning.**

Specifically, we treated $A$, $B$, and the initial state mean and variance as essential parameters, and examined whether the remaining four parameters—$C$, $D$, the process noise variance ($Q$), and the measurement noise variance ($R$)—could be fixed at their mean estimates obtained from the learning model across learning episodes without substantially degrading model fit. This yielded a set of simpler models, which were compared with the learning model using BIC and log-likelihood across all learning episodes.

The results in S2 Table showed that the learning model consistently provided the best overall performance. It achieved the lowest mean BIC (−209.1), the highest mean log-likelihood (124.2), and the largest number and proportion of best-fitting episodes (315 episodes, 17.7%). Although some simpler models, particularly those fixing $Q$ or $C$, showed relatively similar performance, none outperformed the learning model on any of these summary metrics. In contrast, models with multiple fixed parameters showed substantially poorer fits, with progressively higher BIC and lower log-likelihood values.

These findings indicate that the simplified models do not provide an equally adequate description of the learning episodes. Thus, although the learning model is more complex, retaining all eight free parameters is justified and yields the most reliable overall fit for the present dataset.
