# Supplementary material for: To learn or not to learn? Model-based estimation of motor learning in a large, “in the wild”, home rehabilitation database
Source: PLOS Digit Health. 2026 Aug 27;5(8):e0001598. doi: 10.1371/journal.pdig.0001598 (PMC13521380; doi:10.1371/journal.pdig.0001598)
Supplement: Supplementary file 7 [file pdig.0001598.s007.docx]

**S7. Task distribution for users with zero learning and high learning**

To examine whether the classification of non-learners could be influenced by task selection bias, we compared the task distributions of users with zero learning and users with high learning (S8 Fig). Specifically, we tested whether users classified as non-learners were disproportionately concentrated in tasks that were intrinsically less learnable.

The high-learning group was defined as users with percent_learning $\geq$ 71%, which yielded a group size similar to that of the zero-learning group and therefore provided a comparable upper-tail reference group. If task selection bias were driving the non-learner classification, one would expect the zero-learning group to contain a higher proportion of less learnable tasks. However, this was not the case. Instead, users with zero learning engaged in a higher proportion of arm-related tasks (65.6%) than users with high learning (57.6%), even though arm-related tasks were, on average, more learnable than hand-related tasks in our analysis.

To quantify differences in task composition between the two groups, we computed the total variation distance (TVD) between their empirical task distributions. TVD measures the overall dissimilarity between two probability distributions, with larger values indicating greater differences in composition. The observed TVD was 0.141, indicating only a modest difference in task distribution. A permutation test with 10,000 iterations yielded a p-value of 0.082, indicating that this difference did not reach statistical significance at the conventional 0.05 level.

Together, these results provide little evidence that users classified as non-learners were systematically concentrated on more difficult tasks. Thus, task selection bias is unlikely to fully account for the non-learner group identified in the user-level analysis.
