# Supplementary material for: To learn or not to learn? Model-based estimation of motor learning in a large, “in the wild”, home rehabilitation database
Source: PLOS Digit Health. 2026 Aug 27;5(8):e0001598. doi: 10.1371/journal.pdig.0001598 (PMC13521380; doi:10.1371/journal.pdig.0001598)
Supplement: Supplementary file 8 [file pdig.0001598.s008.docx]

**S1. Distributions of episode duration and training sessions.**

**S1 Fig. Distributions of episode duration (in days) and number of training sessions per episode across all (user, task) episodes.** A: Episode duration, defined as the number of calendar days from the first to the last session. B: Number of sessions, defined as days with at least one recorded repetition. Both distributions show substantial variability across episodes. Boxplots below each histogram indicate the median (red line), interquartile range (IQR, box edges), whiskers (1.5×IQR), and outliers (black points). Based on the data preprocessing steps, the resulting 4,661 episodes exhibited a wide range of temporal structures and user engagement patterns, as illustrated in S1 Figure. These distributions highlight the variability in engagement with FitMi over extended timeframes, reflecting the unsupervised and self-directed nature of home rehabilitation.
