# Supplementary material for: To learn or not to learn? Model-based estimation of motor learning in a large, “in the wild”, home rehabilitation database
Source: PLOS Digit Health. 2026 Aug 27;5(8):e0001598. doi: 10.1371/journal.pdig.0001598 (PMC13521380; doi:10.1371/journal.pdig.0001598)
Supplement: Supplementary file 9 [file pdig.0001598.s009.docx]

**S2. Robustness of learning classification**

**S2 Fig. Robustness of episode-level learning classification. Distribution of likelihood ratio (LR) statistics (A), corresponding uncorrected p-values (B), FDR-adjusted p-values (C), and BIC-based learning weights (D) across all 4,661 episodes.** The dashed vertical line in panel A indicates the significance threshold corresponding to p = 0.05 under a chi-square distribution with 6 degrees of freedom ($\chi^{2}=12.59$). Episodes with LR values above this threshold were classified as learning, whereas those below the threshold were classified as non-learning according to this primary likelihood ratio test (LRT)-based analysis. Of the 1,785 episodes significant at the uncorrected threshold of p < 0.05, 1,758 remained significant after Benjamini–Hochberg false discovery rate (FDR) correction, with only 27 episodes changing classification. The distributions of LR statistics, p-values, and BIC-based learning weights all show a polarized pattern, indicating that most episodes provided strong support for either the learning or non-learning model, with relatively few intermediate cases.
