# Supplementary material for: To learn or not to learn? Model-based estimation of motor learning in a large, “in the wild”, home rehabilitation database
Source: PLOS Digit Health. 2026 Aug 27;5(8):e0001598. doi: 10.1371/journal.pdig.0001598 (PMC13521380; doi:10.1371/journal.pdig.0001598)
Supplement: Supplementary file 10 [file pdig.0001598.s010.docx]

**S3.** $\boldsymbol{R}^{\mathbf{2}}$ **for learning vs. non-learning episodes**

**S3 Fig. Comparison of model fit quality (**$R^{2}$**) between learning and non-learning models across episodes.** Each point represents one training episode, with the $x$-axis showing the $R^{2}$ of the non-learning (feedthrough-only) model and the $y$-axis showing the $R^{2}$ of the learning (memory-based) model. Episodes are color-coded based on their classification via likelihood ratio test: blue for episodes classified as “learning” and orange for those classified as “non-learning.” The dashed diagonal line $y=x$ indicates equal fit quality between the two models. Marginal distributions for each group are shown along the top and right axes. Learning episodes generally lie above the diagonal, indicating better fit by the learning model, while non-learning episodes tend to cluster along the diagonal, suggesting similar performance by both models.
