# Supplementary material for: To learn or not to learn? Model-based estimation of motor learning in a large, “in the wild”, home rehabilitation database
Source: PLOS Digit Health. 2026 Aug 27;5(8):e0001598. doi: 10.1371/journal.pdig.0001598 (PMC13521380; doi:10.1371/journal.pdig.0001598)
Supplement: Supplementary file 11 [file pdig.0001598.s011.docx]

**S4. Percentage of ceiling effect for non-learning episodes**

**S4 Fig. Empirical complementary cumulative distribution function (CCDF) of the proportion of observations exceeding the task-specific 95th-percentile performance threshold among non-learning episodes.** For each non-learning episode, we calculated the proportion of observations with performance values above the 95th-percentile threshold. The x-axis shows the cutoff applied to this episode-level proportion, and the y-axis shows the proportion of non-learning episodes with values greater than the corresponding cutoff. The curve declines steeply at lower cutoffs and becomes progressively shallower around 20%, indicating that the number of ceiling-effect episodes is highly sensitive to threshold choice at small values but changes more gradually beyond this point. The dashed vertical line marks the 20% cutoff used to define ceiling-effect episodes in the subsequent analysis.
