# Supplementary material for: To learn or not to learn? Model-based estimation of motor learning in a large, “in the wild”, home rehabilitation database
Source: PLOS Digit Health. 2026 Aug 27;5(8):e0001598. doi: 10.1371/journal.pdig.0001598 (PMC13521380; doi:10.1371/journal.pdig.0001598)
Supplement: Supplementary file 12 [file pdig.0001598.s012.docx]

**S5. Percent of performance variance explained by learning for learning episodes**

**S5 Fig. Distribution of the percentage of performance variance explained by learning across learning episodes.** For each episode classified as “learning,” we computed the percentage of output variance explained by the memory state term $C_{ik}*X_{ik}$ from the learning model. The histogram shows the distribution of this percentage across all episodes, and the horizontal boxplot below summarizes its variability. The median value was 38.8%, with an interquartile range (IQR) of [19.2%, 68.7%], indicating substantial heterogeneity to the extent to which learning contributed to performance improvements.
