# Supplementary material for: To learn or not to learn? Model-based estimation of motor learning in a large, “in the wild”, home rehabilitation database
Source: PLOS Digit Health. 2026 Aug 27;5(8):e0001598. doi: 10.1371/journal.pdig.0001598 (PMC13521380; doi:10.1371/journal.pdig.0001598)
Supplement: Supplementary file 13 [file pdig.0001598.s013.docx]

**S6. Parameter correlation analysis for learning vs. non-learning episodes**

**S6 Fig. Parameter correlation matrices for (A) learning episodes and (B) non-learning episodes.** Cell values and color intensity indicate Pearson correlation coefficients between fitted parameter estimates across episodes. Most parameter pairs showed weak correlations, indicating generally good parameter identifiability. In the non-learning episodes, however, B and D were strongly positively correlated, consistent with reduced identifiability of the learning-related component when $\tau$ is small. A moderate negative correlation between $\tau$ and B is also visible in both groups, reflecting the typical gain–time-constant tradeoff in first-order systems.
