# Supplementary material for: To learn or not to learn? Model-based estimation of motor learning in a large, “in the wild”, home rehabilitation database
Source: PLOS Digit Health. 2026 Aug 27;5(8):e0001598. doi: 10.1371/journal.pdig.0001598 (PMC13521380; doi:10.1371/journal.pdig.0001598)
Supplement: Supplementary file 14 [file pdig.0001598.s014.docx]

**S7. Percentage of long-term learning for learning episodes**

**S7 Fig. Forgetting time constant** $\boldsymbol{\tau}$ **among learning episodes and percentage of long-term learning as a function of varying** $\boldsymbol{\tau}$ **thresholds.** (A) Histogram showing the distribution of the forgetting time constant $\tau$ (in days) among learning episodes. Each bar represents the percentage of learning episodes whose fitted forgetting time constant falls within the corresponding bin. The rightmost bin (> 90) includes all learning episodes with $\tau$ > 90 days. Larger values of $\tau$ indicate slower forgetting and therefore more persistent learning. (B) The percentage of learning episodes classified as long-term learning as a function of the threshold applied to $\tau$. For each threshold value on the x-axis, the y-axis shows the proportion of learning episodes with $\tau$ greater than that threshold, i.e., $\tau$ > threshold. This panel, therefore, illustrates how the estimated fraction of long-term learning episodes changes as the operational definition of “long-term” becomes more stringent. Because the definition of long-term learning based on the forgetting time constant $\tau$ is inherently operational, we examined how the results varied across a range of threshold values. The distribution of fitted $\tau$ values across learning episodes was broad, with shorter time constants more common but a substantial fraction of episodes showing relatively long persistence. As expected, the proportion of episodes classified as long-term decreased smoothly as the threshold increased. The absence of a clear natural breakpoint supports interpreting learning persistence as a continuum and the 30-day threshold as a clinically meaningful, practical cutoff rather than a biologically exact boundary.
