# Supplementary material for: To learn or not to learn? Model-based estimation of motor learning in a large, “in the wild”, home rehabilitation database
Source: PLOS Digit Health. 2026 Aug 27;5(8):e0001598. doi: 10.1371/journal.pdig.0001598 (PMC13521380; doi:10.1371/journal.pdig.0001598)
Supplement: Supplementary file 15 [file pdig.0001598.s015.docx]

**S8. Task distribution for users with zero learning and high learning**

**S8 Fig. Task distribution for users with zero learning (A) and high learning (B; percent_learning** $\boldsymbol{\geq}$ **71%).** Bars indicate the proportion of each task among valid sessions from users practicing at least five tasks. Tasks are color-coded as hand-related (orange) or arm-related (green).
