# Supplementary material for: To learn or not to learn? Model-based estimation of motor learning in a large, “in the wild”, home rehabilitation database
Source: PLOS Digit Health. 2026 Aug 27;5(8):e0001598. doi: 10.1371/journal.pdig.0001598 (PMC13521380; doi:10.1371/journal.pdig.0001598)
Supplement: Supplementary file 16 [file pdig.0001598.s016.docx]

**S9. Parameter recovery via simulation**

**S9 Fig. Model validation of parameter recovery.** Results from simulation-based validation of parameter estimation. True parameters from real data were used to generate noisy synthetic datasets, and the EM algorithm reliably recovered the original values, demonstrating robustness of the estimation procedure. S9 Fig shows the results of our simulation-based validation of parameter recovery. Across episodes, we observed strong agreement between the original parameters (used to generate synthetic data) and the re-estimated values obtained via the EM algorithm. This confirms that the model fitting procedure is robust to realistic levels of noise and variability. Recovery was consistently strong across key parameters, including the learning rate $B$, output gain $C$, and feedthrough term $D$. For the retention time constant $\tau$, we observed higher recovery error at large values of $\tau$. This is expected due to the nonlinear mapping $A=e^{-\frac{1}{\tau}}$: small fluctuations in the estimated $A$ (which is close to 1 when $\tau$ is large) can lead to large numerical changes in $\tau$. Nonetheless, even in these cases, the recovered values remained qualitatively consistent with the true values, preserving the distinction between short-term and long-term learning episodes. Overall, these results validate the accuracy and stability of our parameter estimation approach and support the interpretability of the learning dynamics presented in the main analysis.
