# Supplementary material for: To learn or not to learn? Model-based estimation of motor learning in a large, “in the wild”, home rehabilitation database
Source: PLOS Digit Health. 2026 Aug 27;5(8):e0001598. doi: 10.1371/journal.pdig.0001598 (PMC13521380; doi:10.1371/journal.pdig.0001598)
Supplement: Supplementary file 17 [file pdig.0001598.s017.docx]

**S10. Recovery error for forgetting time constant**

**S10 Fig. Recovery error of the forgetting time constant across true parameter values and numbers of training sessions.** (A) Absolute error versus the true time constant $\tau_{\text{true}}$; (B) Relative error versus $\tau_{\text{true}}$; (C) Absolute error versus the number of training sessions. Each point represents an individual estimate. In (A) and (B), the spread of errors increases as $\tau_{\text{true}}$ becomes larger, reflecting the sensitivity of the transformation from $A$ to $\tau$. In (C), higher numbers of training sessions are generally associated with lower estimation errors, although variability remains due to noise. S9 Fig shows good parameter recovery results overall, but it also indicates that recovery error increases when $\tau$ > 100 days. Therefore, we further analyzed the recovery error for the forgetting time constant τ across different values of $\tau_{\text{true}}$ and different numbers of training sessions. S10 Fig A shows that the absolute error in estimating $\tau$ increases as $\tau_{\mathrm{true}}$ becomes larger. This pattern arises from the nonlinear relationship $A_{ik}=e^{-1/\tau_{ik}}$, where $A_{ik}$ is the memory retention factor derived from the forgetting time constant $\tau_{ik}>0$. When τ is large, $A_{ik}$ approaches 1, so changes in τ produce only very small changes in the implied retention dynamics. As a result, the model becomes less sensitive to differences among large τ values, which amplifies absolute estimation errors in this range. However, as shown in S10 Fig B, the relative error remains fairly stable across different values of $\tau_{\mathrm{true}}$ and rarely exceeds 30%, suggesting that the estimates are reasonably accurate over the simulated range. S10 Fig C further shows that estimation error decreases as the number of training sessions increases, indicating that additional data provide stronger constraints on the model parameters. This is consistent with the interpretation of $A_{ik}$ as a retention factor: more repeated training yields more stable and reliable estimates of the underlying forgetting dynamics.
