# Supplementary material for: To learn or not to learn? Model-based estimation of motor learning in a large, “in the wild”, home rehabilitation database
Source: PLOS Digit Health. 2026 Aug 27;5(8):e0001598. doi: 10.1371/journal.pdig.0001598 (PMC13521380; doi:10.1371/journal.pdig.0001598)
Supplement: Supplementary file 18 [file pdig.0001598.s018.docx]

**S1. Percentage of ceiling effect for non-learning episodes**

**S1 Table. Distribution of episodes across learning and non-learning episodes after ceiling-effect classification.**

| **Group** | **Subgroup** | **Count** | **% within group** | **% of total** |
| --- | --- | --- | --- | --- |
| **Learning**  **(**$\boldsymbol{n=1785}$**)** | Long-term learning  ($\tau\geq$ 30 days) | 797 | 44.6% | 17.1% |
|  | Short-term learning  ($\tau<$ 30 days) | 988 | 55.4% | 21.2% |
| **Non-learning (**$\boldsymbol{n=2876}$**)** | Ceiling effect  ($\geq$ 20% threshold) | 279 | 9.70% | 5.99% |
|  | True non-learning  ($<$ 20% threshold) | 2597 | 90.3% | 55.7% |

Learning episodes were further divided into long-term ($\tau\geq30$ days) and short-term ($\tau<30$ days) learning based on the estimated time constant. Non-learning episodes were further subdivided into ceiling effect, defined as episodes in which at least 20% of observations exceeded the task-specific 95th-percentile threshold, and true non-learning, defined as episodes below this cutoff. “% within group” denotes the percentage within each main category (learning or non-learning), and “% of total” denotes the percentage relative to all episodes. Taking a threshold of 20%, non-learning episodes were further classified into two subgroups: ceiling effect ($\geq$ 20%) and true non-learning (< 20%). The corresponding summary statistics are presented in S1 Table.
