# Supplementary material for: To learn or not to learn? Model-based estimation of motor learning in a large, “in the wild”, home rehabilitation database
Source: PLOS Digit Health. 2026 Aug 27;5(8):e0001598. doi: 10.1371/journal.pdig.0001598 (PMC13521380; doi:10.1371/journal.pdig.0001598)
Supplement: Supplementary file 19 [file pdig.0001598.s019.docx]

**S2.** **Model comparison between learning model vs. simpler models**

**S2 Table. Model comparison for learning episodes between the learning model and simpler models, in which selected parameters were fixed at their mean estimates obtained from the learning model across learning episodes.**

| **Model** | **# Free Parameters** | **Mean BIC** | **Mean LL** | **# Best** | **% Best** |
| --- | --- | --- | --- | --- | --- |
| **Learning** | 8 | -209.1 | 124.2 | 315 | 17.7 |
| **Fix Q** | 7 | -207 | 120.8 | 250 | 14.0 |
| **Fix C** | 7 | -206.5 | 120.5 | 164 | 9.19 |
| **Fix C & Q** | 6 | -192.5 | 111 | 290 | 16.3 |
| **Fix Q & R** | 6 | -137.7 | 83.65 | 49 | 2.75 |
| **Fix R** | 7 | -137.4 | 85.93 | 10 | 0.560 |
| **Fix C & R** | 6 | -136.4 | 82.98 | 42 | 2.35 |
| **Fix D & Q** | 6 | -116.1 | 72.86 | 137 | 7.68 |
| **Fix D** | 7 | -111.6 | 73.04 | 17 | 0.952 |
| **Fix C, Q & R** | 5 | -99.5 | 62.07 | 140 | 7.84 |
| **Fix D & R** | 6 | -94.39 | 61.98 | 10 | 0.560 |
| **Fix D, Q & R** | 5 | -93.59 | 59.12 | 58 | 3.25 |
| **Fix C, D & R** | 5 | -85.93 | 55.28 | 110 | 6.16 |
| **Fix C & D** | 6 | -76.27 | 52.92 | 38 | 2.13 |
| **Fix C, D & Q** | 5 | -65.57 | 45.1 | 101 | 5.66 |
| **Fix C, D, Q & R** | 4 | 200.2 | -90.26 | 54 | 3.03 |

Models were compared by fitting each learning episode separately using EM. In the simpler models, parameters C, D, the process noise variance (Q), and/or the measurement noise variance (R) were fixed at their mean estimates obtained from the learning model across learning episodes, whereas the remaining parameters were estimated freely. “# Free Parameters” denotes the number of free parameters in each model. “Mean BIC” and “Mean LL” denote the BIC and log-likelihood values, respectively, averaged across all episodes. Lower BIC indicates a better trade-off between model fit and complexity. “# Best” and “% Best” denote the number and percentage of learning episodes, respectively, for which a given model achieved the lowest BIC. Overall, **the learning model performed best across all summary metrics, supporting retention of all eight free parameters in the main analysis.**
